# The lamprey habenula provides an extreme example for the temporal regulation of asymmetric development

**DOI:** 10.3389/fcell.2025.1528797

**Published:** 2025-02-06

**Authors:** Lucile Guichard, Ronan Lagadec, Léo Michel, Hélène Mayeur, Michaël Fuentès, Jordan Pain, Noah Heier, Quentin Rougemont, Maria Celina Rodicio, Antón Barreiro-Iglesias, Patrick Blader, Michael Schubert, Sylvie Mazan

**Affiliations:** ^1^ CNRS, UMR7232-Biologie Intégrative des Organismes Marins (BIOM), Observatoire Océanologique, Sorbonne Université, Banyuls-sur-Mer, France; ^2^ CNRS, AgroParisTech, Laboratoire Ecologie Systématique et Evolution, Université Paris-Saclay, Gif-sur-Yvette, France; ^3^ Departamento de Bioloxía Funcional, Facultade de Bioloxía, Universidade de Santiago de Compostela, Santiago de Compostela, Spain; ^4^ Aquatic One Health Research Center (ARCUS), Universidade de Santiago de Compostela, Santiago de Compostela, Spain; ^5^ Molecular, Cellular and Developmental Biology (MCD, UMR5077), Centre de Biologie Intégrative (CBI, FR3743), Université de Toulouse, CNRS, UPS, Toulouse, France; ^6^ Laboratoire de Biologie du Développement de Villefranche-sur-Mer, Institut de la Mer de Villefranche, Sorbonne Université, CNRS, Villefranche-sur-Mer, France

**Keywords:** habenula, asymmetry, lamprey, temporal regulation, Wnt signaling

## Abstract

By their phylogenetic position and their marked epithalamic asymmetries, lampreys are relevant models for understanding the formation and evolution of this trait across vertebrates. In this study, we use a transcriptomic approach to identify novel signature markers to characterize the highly asymmetric, bipartite organization of habenulae in lampreys. Lamprey habenulae are subdivided into two complementary subdomains related, respectively, to the lateral/ventral and the medial/dorsal habenulae of jawed vertebrates: a dorsal, right-restricted subdomain and a bilateral subdomain that includes the left habenula as well as its ventral right counterpart. Analysis of the formation of the lamprey habenula at prolarval and larval stages using a combination of morphological, immunohistochemical, and *in situ* hybridization approaches highlights a marked asymmetric temporal regulation. The dorsal right subdomain forms and already expresses all identified signature markers in prolarval stages. In contrast, the left and ventral right subdomain appears significantly later, with the first indication of neuronal identity elaboration in these territories being observed in larval stages. As in gnathostomes, Wnt signaling may be involved in the regulation of this unique, asymmetric mode of development, since β-catenin shows asymmetric and highly dynamic nuclear distributions both in neural progenitors and differentiated neuronal precursors of the two habenular subdomains. These data confirm the importance of lampreys to unravel the developmental logic underlying the recurrence and variation of habenular asymmetries in vertebrates and pave the way for future functional analyses.

## 1 Introduction

The habenula is a bilateral epithalamic structure that forms a key node in neuronal circuits connecting the basal forebrain with different midbrain and brainstem nuclei (Ables et al., 2023; Beretta et al., 2012). Based on projection analyses and molecular characterizations in different vertebrate taxa, including mammals and teleosts, but also cyclostomes (or jawless vertebrates) and chondrichthyans (or cartilaginous fishes), vertebrate habenulae have been shown to share a conserved bipartite organization (medial/lateral or dorsal/ventral in jawed vertebrates) (Amo et al., 2010; Grillner et al., 2018; Stephenson-Jones et al., 2012). A remarkable feature of this structure in humans and many other vertebrates is that it displays asymmetries between the left and right sides (Abuduaini et al., 2023; Ahumada-Galleguillos et al., 2016; Concha and Wilson, 2001; Hitti et al., 2022). The biological functions of these asymmetries have been assessed in the zebrafish, where they regulate the integration of sensory cues and adaptive responses to the environment (Chen et al., 2019; Dreosti et al., 2014; Duboué et al., 2017; Facchin et al., 2015). As expected from structures processing input from different ecological contexts, habenular asymmetries vary considerably across vertebrates not only in their degree but also in their nature, with variations reported in neuronal identities and projections, as well as in the broad organization of habenular subdomains (Concha and Wilson, 2001; Stephenson-Jones et al., 2012). Despite their biological relevance, the evolutionary origin of habenular asymmetries in vertebrates and the ontogenetic variations underlying their diversification have remained for long largely unexplored, with most studies focusing on teleosts, primarily the zebrafish (Michel et al., 2022; Roussigné et al., 2012; Signore et al., 2009; Villalón et al., 2012). Recently, however, a transcriptomic characterization of habenular asymmetries and an analysis of the mechanisms underlying their formation in a cartilaginous fish (or chondrichthyan), the small spotted catshark *Scyliorhinus canicula*, provided new insights into the evolution of habenular development (Lanoizelet et al., 2024). A systematic comparison of the catshark with species occupying key phylogenetic positions in the vertebrate tree suggested an ancient origin of asymmetries in the lateral habenula. This study also highlighted Wnt signaling as a candidate mechanism involved in both asymmetry formation and diversification across vertebrates. As in the zebrafish, a left repression of Wnt activity is thus observed in the catshark, in line with the hypothesis of an ancient involvement of Wnt signaling in habenular asymmetry formation (Guglielmi et al., 2020; Hüsken et al., 2014; Lanoizelet et al., 2024). However, ancestral Wnt-dependent cellular mechanisms and regulatory programs remain unclear in view of major differences between the zebrafish and the catshark. Wnt signaling thus operates in different cellular contexts (lateral habenula in the catshark, dorsal habenula in the zebrafish), via distinct cellular mechanisms (neuronal identity choices in post-mitotic precursors in the catshark, control of neurogenesis timing in neural progenitors in the zebrafish), and downstream of different developmental regulations (Nodal- and parapineal-dependent, respectively, in the catshark and the zebrafish) (Guglielmi et al., 2020; Lagadec et al., 2015; Lanoizelet et al., 2024; Powell et al., 2024).

Genome-wide characterizations of habenular asymmetries and detailed analyses of their mechanisms of formation are required in a broader sampling of species to obtain a comprehensive picture of their nature in ancestral vertebrates and to clarify the functional evolution of Wnt signaling in their formation. Lampreys are crucial species in this respect. As members of the cyclostomes, the sister group of gnathostomes, they occupy an important phylogenetic position to reconstruct ancestral vertebrate states (Shimeld and Donoghue, 2012). They are also endowed with marked habenular asymmetries that exhibit both conserved and divergent features when compared to gnathostomes, which makes lampreys a relevant model for studying the evolution of these asymmetries. Analyses of efferent projection patterns, supported by expression profiles of a limited number of subdomain markers, suggest that the right lateral habenula of the catshark corresponds to the right dorsal habenula of the river lamprey. This work has also pointed to a complete absence of a lateral component in the lamprey left habenula, a characteristic never observed in gnathostomes (Grillner et al., 2018; Stephenson-Jones et al., 2012). The molecular mechanisms underlying the formation of habenular asymmetries during lamprey development remain largely elusive. The only study on this subject revealed an ancestral dependence on Nodal signaling, shared by the river lamprey and the catshark, but absent in zebrafish (Lagadec et al., 2015). Genome-wide characterizations of habenular asymmetries as well as cellular and molecular analyses of their mechanisms of formation in the lamprey are important to establish the lamprey as a reference for comparisons with gnathostome model organisms and thus to reconstruct ancestral vertebrate traits. As a first step towards further functional analyses of habenular asymmetry formation in lampreys, we have used a transcriptomic approach to provide an unbiased identification of habenular asymmetries in the river lamprey (*Lampetra fluviatilis*). Using asymmetric markers identified in our transcriptomic screen, we describe the elaboration of habenula subdomain organization in lampreys at prolarval and larval stages. Our results provide a refined view of the subdomain organization of the lamprey habenula and highlight a remarkable asymmetry in the temporal regulation of its formation.

## 2 Materials and methods

### 2.1 Animals and tissue collection

Adult river lamprey (*L. fluviatilis*) specimens, collected during their upstream migratory phase in the Dordogne river, were purchased from professional fishermen. The animals were transported to the Observatoire Océanologique in Banyuls-sur-Mer, France, and maintained at 12°C in oxygenated, filtered fresh water until sexual maturation. Oocytes and sperm were obtained from mature animals by gentle stripping and mixed in Petri dishes for fertilization. Embryos and prolarvae were maintained at 12°C in oxygenated fresh water and staged according to (Tahara, 1988). Young river lamprey larvae with sizes between 0.8 and 1.3 cm were maintained under the same conditions and fed by addition of Tetra Micro Granules (Tetra, Blacksburg, VA, United States). Older larvae (sizes between 3.5 and 7.0 cm) from two other lamprey species, the sea lamprey *Petromyzon marinus* and the brook lamprey *Lampetra planeri*, were, respectively, collected in the river Ulla (Galicia, Spain) with permission from the Xunta de Galicia and in the river Oir (Normandie, France) with permission from the préfecture de la Manche. Ethical review and agreement were not required for analyses of these specimens according to national and European regulations because their study only involved analyses of non-feeding prolarvae or brain tissue collection from euthanized adults and larvae. All prolarvae, larvae, and adults were euthanized by immersion in an overdose of buffered tricaine solution (>1 g/L).

### 2.2 RNA isolation, library construction, and sequencing

Left and right habenula explants were manually dissected from brains collected from euthanized adult river lampreys and stored in TRI reagent (T9424, Sigma-Aldrich, Saint-Louis, MO, United States) at −20°C until RNA extraction. Three left pools, each containing 4 left habenulae from two females and two males and three right pools, containing the corresponding 4 right habenulae, were prepared from these explants. Total RNA was isolated using the NucleoSpin RNA Clean-up XS kit (740903, Macherey-Nagel, Düren, Germany). RNA quantities and integrity indexes (RINs) were assessed prior to Illumina library construction using a Bioanalyzer 2100 (Agilent Technologies, Santa Clara, CA, United States). For each pool analyzed, the smallest RIN measured was 9.9. Subsequently, 50 ng of total RNA from each pool was used to isolate mRNAs using the NEBNext Poly(A) mRNA Magnetic Isolation Module (E7490, NEB, Ipswich, MA, United States). Libraries were then prepared using the NEBNext Ultra II RNA Library Prep kit for Illumina (E7770, NEB, Ipswich, MA, United States). Each library was validated using a Bioanalyzer 2100, quantified using the QuantiFluor dsDNA system with a Quantus Fluorometer (E2670, Promega, Madison, WI, United States), and equal amounts of each library were pooled. Paired-end 100 base-pair sequencing was performed on a DNBSEQ-T7 (Shenzhen, Guangdong, China) generating about 1 billion reads (The NCBI identifier for this dataset is SRS233376, as indicated in Supplementary Table S2).

### 2.3 Read mapping and statistical analysis

In the absence of an annotated genome of the river lamprey, we generated a reference database for read mapping (Supplementary Table S1). This was done by clustering of the datasets listed in Supplementary Table S2. To do so, we used the DRAP 1.92 runDrap pipeline (Cabau et al., 2017). The pipeline used Trinity (Grabherr et al., 2011) for normalization and Oases (Schulz et al., 2012) with kmers 37, 47, 57, and 63 for assembly, with subsequent merging using the DRAP runMeta pipeline. We mapped the original reads onto the resulting assembly using bowtie2 (Langmead and Salzberg, 2012) and used Corset (Davidson et al., 2014) and SuperTranscripts (Davidson et al., 2017) on the resulting alignment file to merge putatively redundant transcripts and putative splice site variants, yielding transcripts representative of all splicing variants of their corresponding genes. For the transcriptomic analysis of asymmetries, reads were pseudo-mapped onto this database of reference gene models and pseudo-counted using a k-mer quantification method, kallisto (Bray et al., 2016). Contigs exhibiting statistically significant count differences between the left and the right habenulae were identified using the Wald test (q-value threshold 5E-02) implemented in sleuth (Pimentel et al., 2017) and annotated by similarity search against cyclostome sequences from Swissprot. We refer to the corresponding genes as left- or right-enriched.

### 2.4 *In situ* hybridization (ISH) of sections

Whole lamprey specimens (prolarvae and 0.8–1.3 cm larvae) or dissected brains (3.5–7.0 cm larvae and adults) were fixed, dehydrated, and stored at −20°C until paraffin embedding and sectioning (section thickness: 5–10 μm). ISH of paraffin sections was carried out using digoxigenin-labeled antisense RNA probes, transcribed *in vitro* from synthetic gene fragments using a standard protocol (Derobert et al., 2002). Following ISH, nuclei were counterstained using Nuclear Fast Red solution (N3020, Sigma-Aldrich, Saint-Louis, MO, United States) and mounted in Eukitt (03989, Sigma-Aldrich, Saint-Louis, MO, United States). Brain sections were imaged with a Zeiss Axioscope 5 (Carl Zeiss Microscopy, Germany) equipped with a ZEISS Axiocam 208 color camera and the Zeiss ZEN Blue software (version 3.7.4). Probe sequences are listed in Supplementary Table S3. They were inferred from river lamprey sequences but also used for ISH of sections from sea lamprey and brook lamprey, the high level of sequence similarity between the three species allowing extensive cross-hybridizations, as previously reported (Lagadec et al., 2015).

### 2.5 Immunohistochemistry (IHC) of sections

After epitope unmasking, paraffin sections were subjected to fluorescent IHC as previously described (Lagadec et al., 2018), with the following modification: for the detection of β-catenin antibody, an additional signal amplification was conducted using the TSA plus Cyanine 5 kit (NEL745001KT, Akoya Biosciences, Menlo Park, CA, United States) following the supplier’s instructions. Antibodies and the concentrations used in this study are listed in Supplementary Table S4. Brain sections were imaged with an inverted Leica SP8 microscope (Leica Microsystems Inc., Wetzlar, Germany) equipped with a SuperK EXTREME white laser source (NKT Photonics A/S, Birkerød, Denmark) and a Leica Hybrid Detector. Images were processed using ImageJ.

### 2.6 TUNEL assays on sections

Apoptosis was assessed on paraffin-embedded sections with the *In Situ* Cell Death Detection kit (11684809910, Roche, Basel, Switzerland) as previously described (Lagadec et al., 2018). Briefly, slides were pretreated in 0.1 M citrate buffer (pH6.0) with a 350 W microwave irradiation for 5 min and incubated in the TUNEL reaction mixture for 1 h at 37°C prior to DAPI nuclear staining. For positive controls, a DNase I treatment (1000 U/mL) was carried out for 10 min at room temperature prior to the labeling procedure. For negative controls, slides were treated as described above, except that terminal transferase was omitted in the reaction.

## 3 Results

### 3.1 Transcriptomic analysis of asymmetries in river lamprey habenulae

To obtain an unbiased characterization of asymmetries in river lamprey habenulae, we carried out a transcriptomic comparison between their right and left moieties. To do so, we constructed barcoded Illumina libraries using total RNA extracted from pools of manually dissected left and right habenula explants. Library sequencing from three replicates for each side led to a total of 994 million reads, which were mapped against an annotated reference gene model database (Supplementary Table S1). Their statistical analysis resulted in the identification of 11799 contigs differentially expressed between the left and the right sides, 6174 left-enriched and 5625 right-enriched (Supplementary Figure S1A; Supplementary Table S5). Genes previously shown to be asymmetrically expressed, such as *Prox1a* (Cluster-16156.0; q-value = 5.9e-50; fold change = 26.8 to the right), *Prox1b* (Cluster-16156.1; q-value = 3.0e-62; fold change = 7.73 to the right) or *Kctd12* (Cluster-4041.6113; q-value = 1.0e-33; fold change = 3.03 to the left) were retrieved in this analysis, with the expected laterality. To obtain a spatial characterization of asymmetries, we conducted ISH analyses on adult habenula sections for a total of 29 genes (15 left-enriched and 14 right-enriched), selected among those exhibiting the highest statistical support for left- or right-enrichment in the transcriptomic analysis (Supplementary Figure S1A). Regionalized asymmetric profiles were obtained in all cases except for 6 genes (Supplementary Table S3), and they confirmed the expression laterality predicted by the transcriptomic analysis (Supplementary Figure S1B–X). All left-enriched genes exhibiting a regionalized expression share a broad territory in the left habenula (Supplementary Figures S1B–L), with two of them being only expressed in a dorso-anterior subdomain (*Gucy2g* and *Scgn*) (Supplementary Figure S1C,D) and with six of them exhibiting additional right-sided ventral territories (*Rock2*, *Nwd2*, *Necab1*, *Prkar2a*, *Adcy2*, and *Gpr26*) (Supplementary Figures S1F,G,H,I,J,L). Similarly, all right-enriched genes with a regionalized expression show largely overlapping, right-restricted expression territories (Supplementary Figures S1M–X).

### 3.2 Subdomain organization of adult river lamprey habenulae

**FIGURE 1:**
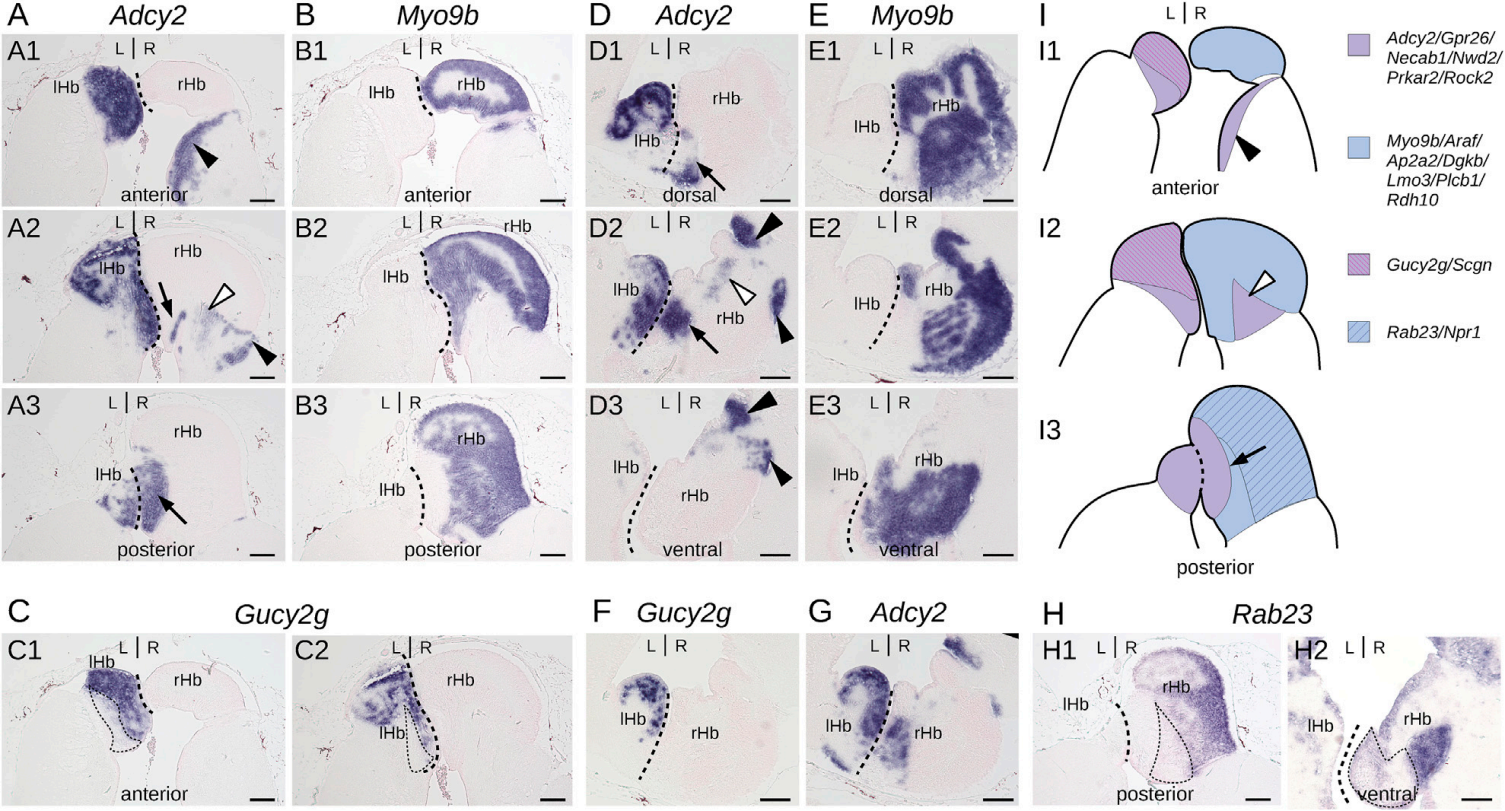
Subdomain organization of adult lamprey habenulae. **(A–C,H1)** show transverse sections and **(D–G,H2)** horizontal sections of adult river lamprey habenulae after *in situ* hybridization with probes for *Adcy2* **(A,D, G)**, *Myo9b* **(B, E)**, *Gucy2g* **(C, F)**, and *Rab23* **(H)**. **(A–C,H1)** are sections from the same specimen, same for **(D–G,H2)**. **(A1–A3)** show successive sections from anterior to posterior, same for **(B1–B3)**. **(D1–D3)** show successive sections from dorsal to ventral, same for **(E1–E3)**. **(A1, B1, C1)** are adjacent sections, same for **(A2, B2, C2)**, for **(B3)** and **(H1)**, for **(D2)**, **(F)**, and **(G)** as well as for **(E3)** and **(H2)**. Dashed lines show the boundary between left and right habenulae. Thin dotted lines in **(C1, C2)** and in **(H1, H2)** respectively delimit a *Adcy2*-positive subdomain negative for *Gucy2g* and a *Myo9b*-positive subdomain negative for *Rab23*. Thin arrows as well as black and white arrowheads point to discrete ventral subterritories expressing *Adcy2* in the right habenula. **(I)** are schemes showing the subdomain organization of habenulae as observed on transverse sections from anterior **(I1)** to posterior **(I3)**. Signature genes for each color are shown on the right based on profiles shown in this figure and in Supplementary Figure S1B-X. Abbreviations: L, left; R, right; lHb, left habenula; rHb, right habenula. Scale bar = 100 μm.

To more precisely describe the subdomain organization of lamprey habenulae, we focused on four markers exhibiting distinct territories with sharp expression boundaries, *Gucy2g*, *Adcy2*, *Myo9b,* and *Rab23*. The relative organization of their respective territories was examined on adjacent sections of the same specimens, along transverse and horizontal planes (Figure 1; Supplementary Figure S2). *Adcy2* and *Myo9b* show mutually exclusive, complementary territories, altogether spanning the whole left and right habenulae (compare Figures 1A,B,D,E; Supplementary Figures S2B,C). *Myo9b* is strictly restricted to the right habenula, with a broad expression territory located dorsally at anterior levels and laterally at posterior ones. This territory excludes three well-delimited, right-sided *Adcy2*-positive cell populations, occupying, respectively, a ventro-lateral position (Figures 1A1,D2,D3; Supplementary Figures S2B1–3), a ventro-medial location in a tract-rich zone (Figures 1A2,D2; Supplementary Figures S2B2–4), and a medial position, adjacent to the midline, at posterior levels (Figures 1A3,D1–3; Supplementary Figures S2B4,5). *Adcy2* expression spans the whole left habenula in addition to these three right-sided cell populations. Analysis of *Gucy2g* and *Rab23* highlight further partitioning of the left habenula and of the *Myo9b*-positive territory in the right habenula. *Gucy2g* marks a dorso-anterior subdomain of the left habenula (Figures 1C,F; Supplementary Figure S2A). The *Rab23* territory is superimposable on that of *Myo9b* in the right habenula, excepting a medial posterior subdomain that is devoid of *Rab23* expression (Figure 1H; Supplementary Figures S2,D5).

### 3.3 Major morphological asymmetries in developing lamprey habenulae

**FIGURE 2:**
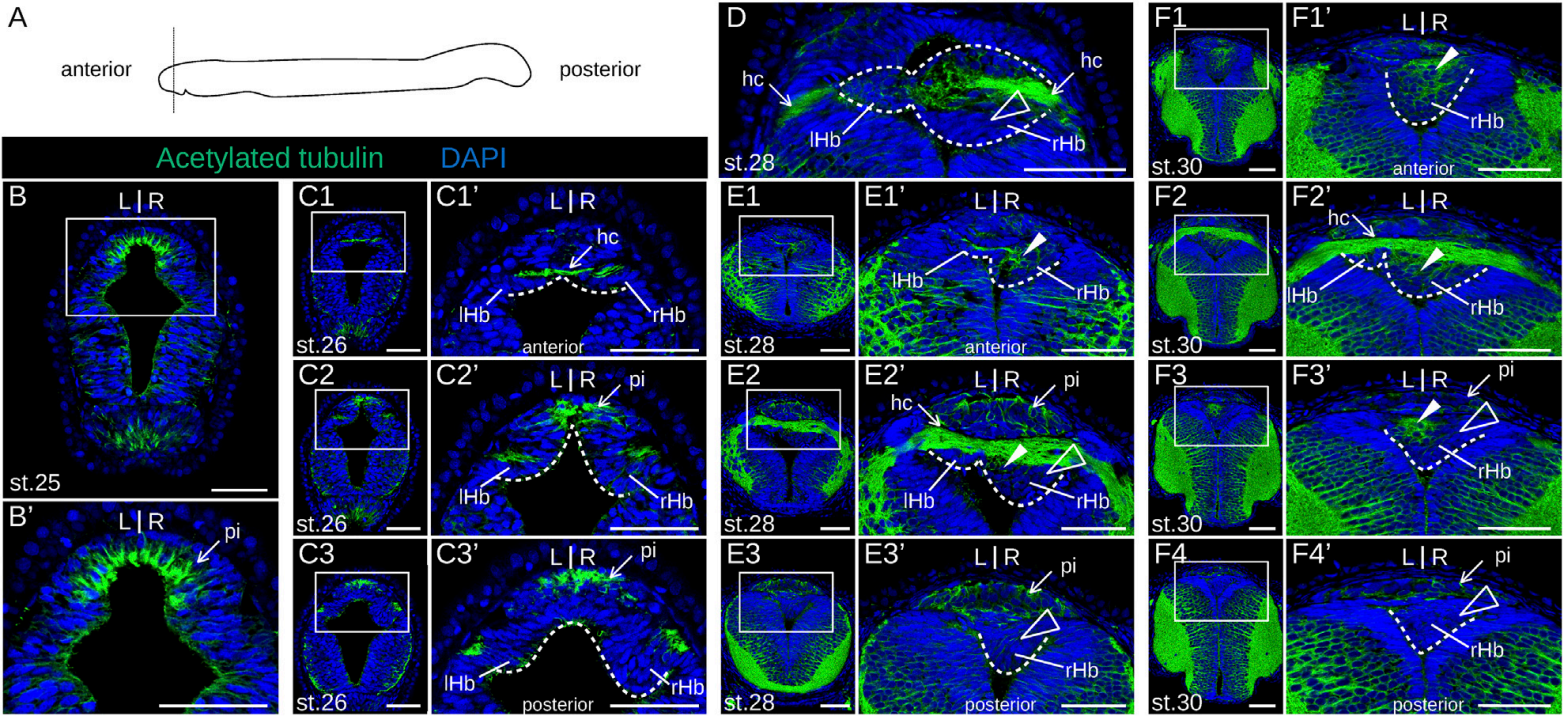
Morphology of lamprey developing habenulae at prolarval stages. **(A)** is a scheme showing the section plane used, orthogonal to the anterior-posterior axis. **(B)**, **(C1-3)**, **(E1-3)**, and **(F1-4)** show transverse sections of developing river lamprey habenulae at prolarval stages 25, 26, 28, and 30, respectively, after immunohistochemistry with an antibody directed against acetylated tubulin (green) and DAPI staining (blue). **(D)** is a horizontal section at stage 28 with the same labeling. **(C1–C3)** show successive sections of the same specimen from anterior to posterior, same for **(E1–E3)**, and for **(F1–F4)**. **(B′,C1′–C3′,E1′–E3′,F1′–F4′)** show higher magnifications of the habenula region boxed respectively in **(B,C1–C3,E1–E3,F1–F4)**. Dashed lines delimit the forming habenulae, the left one being restricted to a thin cell population in contact with the habenular commissure at these stages. White arrowheads point to neuropil-rich territories in the right habenula, as assessed from acetylated tubulin signals. Empty arrowheads point to territories devoid of acetylated tubulin labeling. Abbreviations: L, left; R, right; lHb, left habenula; rHb, right habenula; hc, habenular commissure; pi, pineal field; st., stage. Scale bar = 50 μm.

**FIGURE 3:**
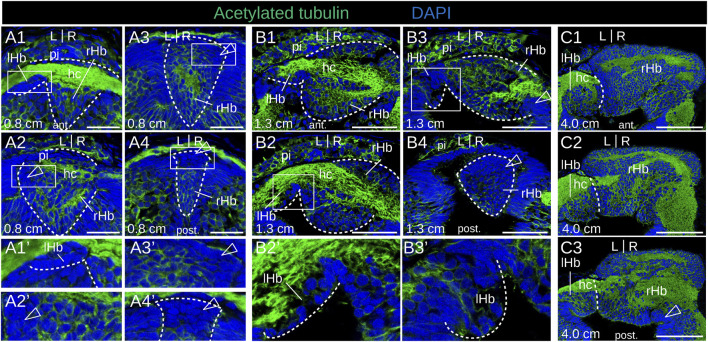
Morphology of lamprey developing habenulae at larval stages. **(A1–4)**, **(B1–4)**, and **(C1–3)** are transverse sections of developing habenulae in 0.8 cm, 1.3 cm and 4.0 cm larvae, respectively, after immunohistochemistry with an antibody directed against acetylated tubulin (green) and DAPI staining (blue). **(A1–4)** and **(B1–4)** were obtained from river lamprey larvae, while **(C1–3)** were obtained from a brook lamprey larva. **(A1–A4)** show successive sections of the same specimen from anterior to posterior, same for **(B1–B4)**, and for **(C1–C3)**. **(A1′–A4′,B2′–B3′)** show higher magnifications of the habenula region boxed respectively in **(A1–A4,B2–B3)**. Dashed lines delimit the forming habenulae in **(A1–A4,A1′,A4′,B1–B4)** and show the boundary between left and right habenulae in **(C1–C3)**. Empty arrowheads point to territories devoid of acetylated tubulin labeling. Abbreviations: L, left; R, right; lHb, left habenula; rHb, right habenula; hc, habenular commissure; pi, pineal field; ant., anterior; post., posterior. Scale bar = 25 μm in **(A1–4)**, 50 μm in **(B1–4)**, and 100 μm in **(C1–3)**.

We next addressed the developmental sequence leading to the elaboration of asymmetries in the river lamprey. To do so, we first generated a morphological reference, using IHC on serial sections with an antibody directed against acetylated tubulin (Figure 2). Habenular evaginations first become visible at stage 25, as previously described (Lagadec et al., 2015), ventral to an acetylated tubulin-positive, neuropil-rich zone corresponding, by its location, to the pineal field (Figure 2B1,B1′). At this stage, the right evagination already appears larger than the left one. At stage 26, the habenular commissure becomes visible (Figure 2C1,C1′). The difference in size between left and right habenulae is more conspicuous in the posterior part of the structure (Figures 2C2,C2′,C3,C3′). Acetylated tubulin signals are maintained in the pineal field but are undetectable in the habenula, except at the level of the habenular commissure. At stage 28, the left habenula is restricted to a thin layer of cells, located ventrally to the habenular commissure (Figure 2E1',E2′), while the right habenula expands towards the left side, occupying most of the neural tube lumen posteriorly (Figures 2E1'–E3′). At this stage, acetylated tubulin is detected anteriorly in the right habenula, with the neuropil being completely absent from the posterior part of the right habenula (Figures 2D,E1'–E3′). These broad characteristics are maintained at stage 30, with a clear partitioning of the right habenula into an expanding anterior, neuropil-rich territory (Figures 2F1′,F2′,F3′) and a posterior territory devoid of acetylated tubulin signal (Figures 2F3′,F4′). Of note, at the level of the habenular commissure, most of the right habenula is located ventrally to the commissure with the exception of a few dorsal cells adjacent to the morphologically distinct pineal rudiment (Supplementary Figures S3A,A1). A change in morphology and size of the left habenula was only observed at the earliest larval stage analyzed (in 0.8 cm larvae). At this stage, dispersed inner cells accumulate between the habenular commissure and the ventricular cell layer (Figure 3A1,A1′). The whole right habenula exhibits acetylated tubulin labeling, except at a posterior dorsal level at the transition to the adjacent thalamus (Figures 3A3,A3′,A4,A4′). In 1.3–6.2 cm larvae, the left habenula has grown posteriorly and ventrally relative to the habenular commissure (Figures 3B2,B3,B2′,B3′; Supplementary Figures S3B–D). On the right, two ventral lobes, separated by a constriction most visible anteriorly, and distinct cell populations, differing by the density of their nuclei, become detectable in 4.0–6.2 cm larvae (Figures 3C1–C3; Supplementary Figures S3B–D). As observed in adults (Lanoizelet et al., 2024), a dense cell organization prevails dorsally in the right habenula of 6.2 cm larvae. In contrast, at ventral levels, cells appear more dispersed and are characterized by larger nuclei, similar to the cell organization observed in the left habenula (Supplementary Figures S3B1–B3,C1). Cells devoid of acetylated tubulin signal with a neuroepithelial organization are restricted to a small lateral and ventral territory of the right habenula in 1.3–6.2 cm larvae (Figures 3B4,C3; Supplementary Figures S3B–D).

### 3.4 Highly asymmetric proliferation-differentiation patterns in developing lamprey habenulae

**FIGURE 4:**
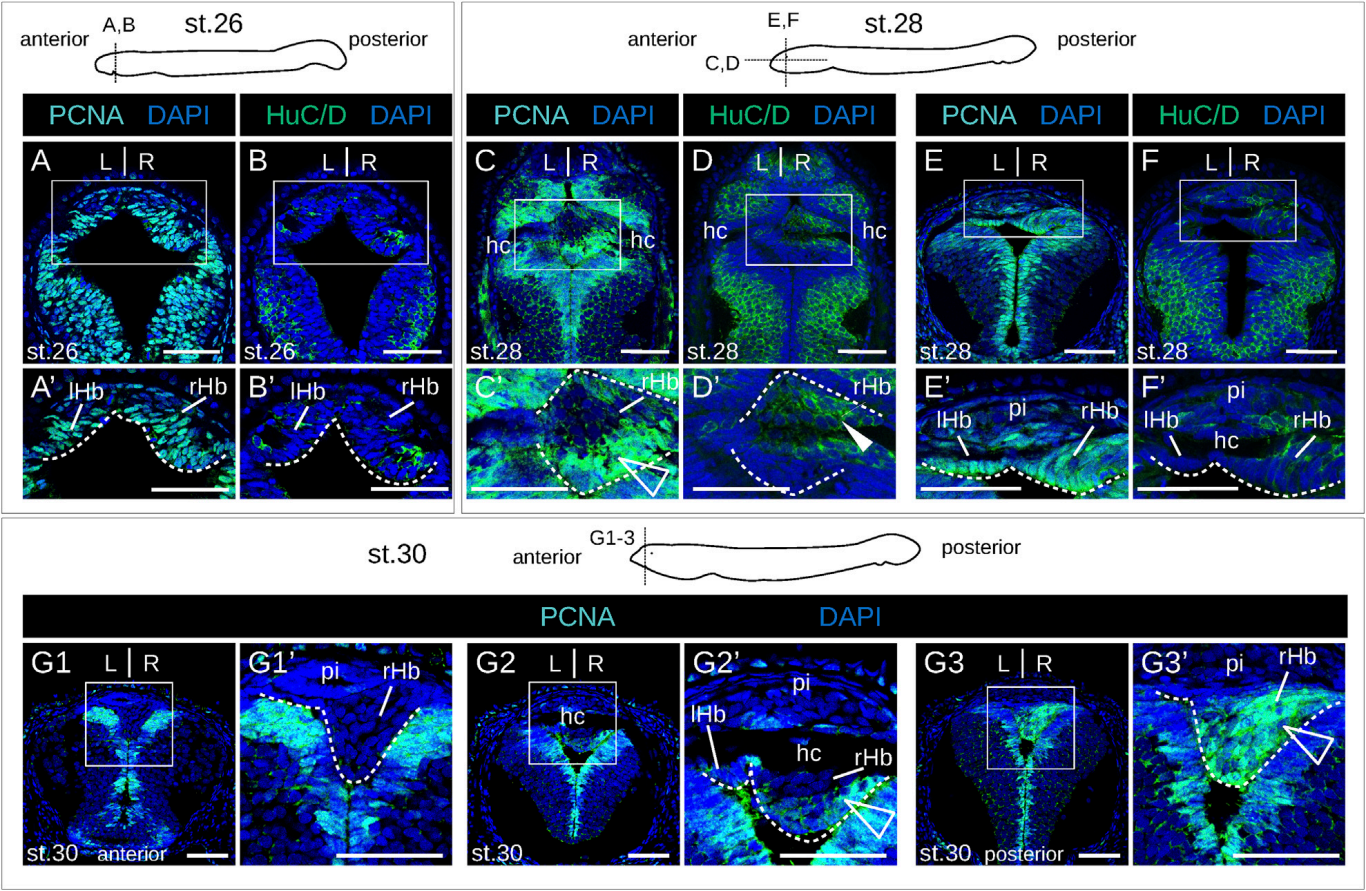
Proliferation-differentiation pattern in developing lamprey habenulae at prolarval stages. **(A,C,E,G1-3)** and **(B,D,F)** are sections of developing river lamprey habenulae at prolarval stages 26 **(A, B)**, 28 **(C–F)**, and 30 **(G1–G3)**, after immunohistochemistry with antibodies directed against either PCNA (green) **(A,C,E,G1–G3)** or HuC/D (green) **(B,D, F)** and DAPI staining (blue). Section planes are shown on schemes above the photographs **(A,B,E,F,G1-3)**: transverse planes; **(C, D)**, horizontal planes. **(G1–G3)** show sections of the same specimen from anterior to posterior. **(A′–F′, G1′–G3′)** are higher magnifications of the habenula region boxed respectively in **(A–F, G1–G3)**. White arrowheads point to anterior HuC/D-positive territories, empty arrowheads highlight posterior PCNA-positive territories in right habenulae at stages stage 28–30. Abbreviations: L, left; R, right; lHb, left habenula; rHb, right habenula; hc, habenular commissure; pi, pineal field; st., stage. Scale bar = 50 μm.

**FIGURE 5:**
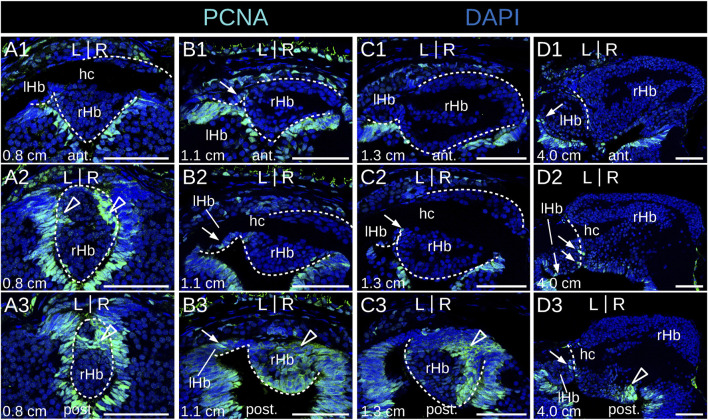
Identification of neural progenitors in developing lamprey habenulae at larval stages. **(A1-3)**, **(B1-3)**, **(C1-3)**, and **(D1-3)** are transverse sections of developing habenulae in respectively 0.8 cm, 1.1 cm, 1.3 cm, and 4.0 cm larvae, after immunohistochemistry with an antibody directed against PCNA (green) and DAPI staining (blue). **(A1–A3)** show sections of the same specimen from anterior to posterior, same for (B1–**B3)**, for **(C1–C3)**, and for **(D1–D3)**. Sections were obtained from river lamprey specimens in **(A1**–**A3,B1**–**B3,C1**–**C3)** and from a brook lamprey specimen in **(D1–D3)**. Empty arrowheads point to posterior PCNA-positive territories in the right habenula, thin arrows point to dispersed PCNA-positive cells in the left habenula. Abbreviations: L, left; R, right; lHb, left habenula; rHb, right habenula; hc, habenular commissure; ant., anterior; post., posterior. Scale bar = 50 μm.

No evidence of apoptosis was observed using a TUNEL assay at prolarval stages (Supplementary Figure S4). To identify neural progenitors and gain insights into the mode of habenula growth, we used IHC with an antibody directed against PCNA (proliferating cell nuclear antigen). IHC using an antibody directed against HuC/D (RNA-binding proteins of the Elav family) was also carried out at prolarval stages to identify newborn neurons. At stage 26, most habenular cells show nuclear signals for PCNA (Figure 4A), but only a few cells are positive for HuC/D, which is more abundant on the right than on the left side and more consistently observed at lateral locations (Figure 4B; Supplementary Figure S5). Very different PCNA profiles are observed between left and right habenulae at subsequent stages. In the right habenula, at stage 28, PCNA nuclear signals become restricted to ventral and posterior territories (Figures 4C,C′) and correlate with those devoid of neuropil as assessed by acetylated tubulin labeling. HuC/D-positive cells are preferentially located anteriorly, at the same location as the neuropil-rich territories expressing acetylated tubulin (Figures 4D,D′). In stage 30 prolarvae, a dense population of PCNA-positive nuclei in territories devoid of neuropil is maintained in the posterior part of the right habenula (Figures 4G1–G3,G1′-G3′). In the left habenula, most cells show nuclear PCNA signals and are negative for HuC/D at stage 28 (Figures 4E,E',F,F′). At stages 29–30, HuC/D-expressing cells become visible medially at the level of the habenular commissure, concomitantly with a loss of nuclear signals of PCNA at this location (Figure 4G2,G2′; Supplementary Figure S6A-C). Concerning larval stages, in 0.8 cm larvae, a strong nuclear PCNA signal is observed posteriorly and dorsally, at the level of the transition to presumptive thalamic territories (Figures 5A1–A3). This dense population is gradually displaced laterally in the right habenula during larval growth (Figures 5B1–B3,C1-C3) and only occupies a restricted posterior ventral territory of the right habenula in 4.0 cm larvae (Figure 5D3). At later larval stages, PCNA-positive nuclei are maintained at this level but become interspersed with PCNA-negative nuclei (Supplementary Figures S6D1–3,D5,E1–3). In the left habenula, at all larval stages examined, ventricular expression of PCNA in left habenulae is generally weak and restricted to dispersed nuclei (Figures 5B1–3,C2,D1-3; Supplementary Figures S6D1–4,E2–3).

### 3.5 Expression timing of subdomain markers in developing lamprey habenulae

**FIGURE 6:**
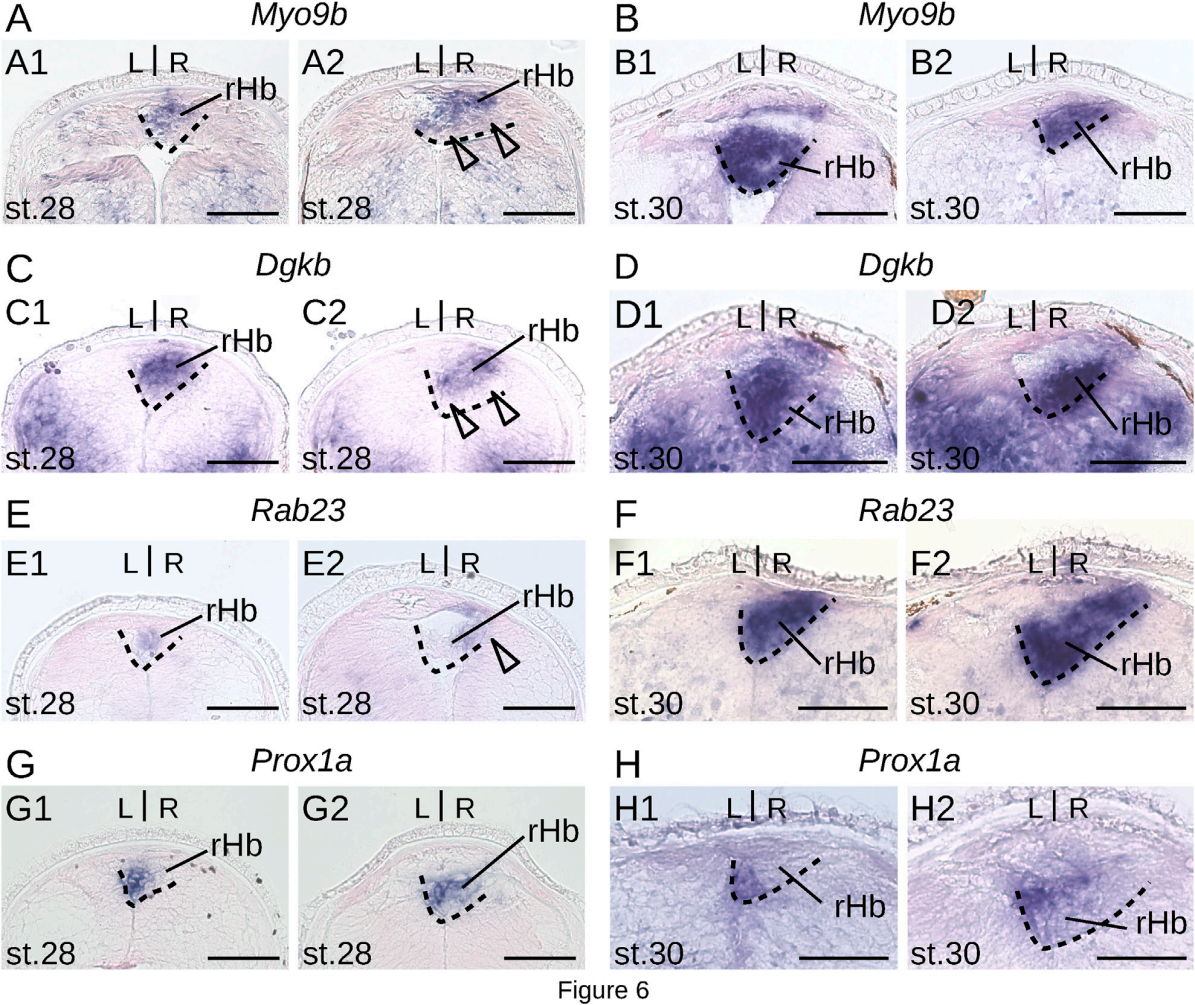
Expression of markers of the dorsal right habenula during lamprey prolarval stages. **(A,C,E, G)** and **(B,D,F, H)** are transverse sections of developing habenulae in respectively stage 28 and stage 30 river lamprey prolarvae, after *in situ* hybridization with probes for *Myo9b* **(A, B)**, *Dgkb* **(C, D)**, *Rab23* **(E, F)** and *Prox1a* **(G, H)**. **(A1–H1)** are from the same specimen as **(A2–H2)** and are located anterior to **(A2–H2)**. Dashed lines delimit the right habenula. Empty arrowheads point to negative territories for the gene analyzed, corresponding by their location to PCNA-positive cell populations. Abbreviations: L, left; R, right; lHb, left habenula; rHb, right habenula; st., stage. Scale bar = 50 μm.

**FIGURE 7:**
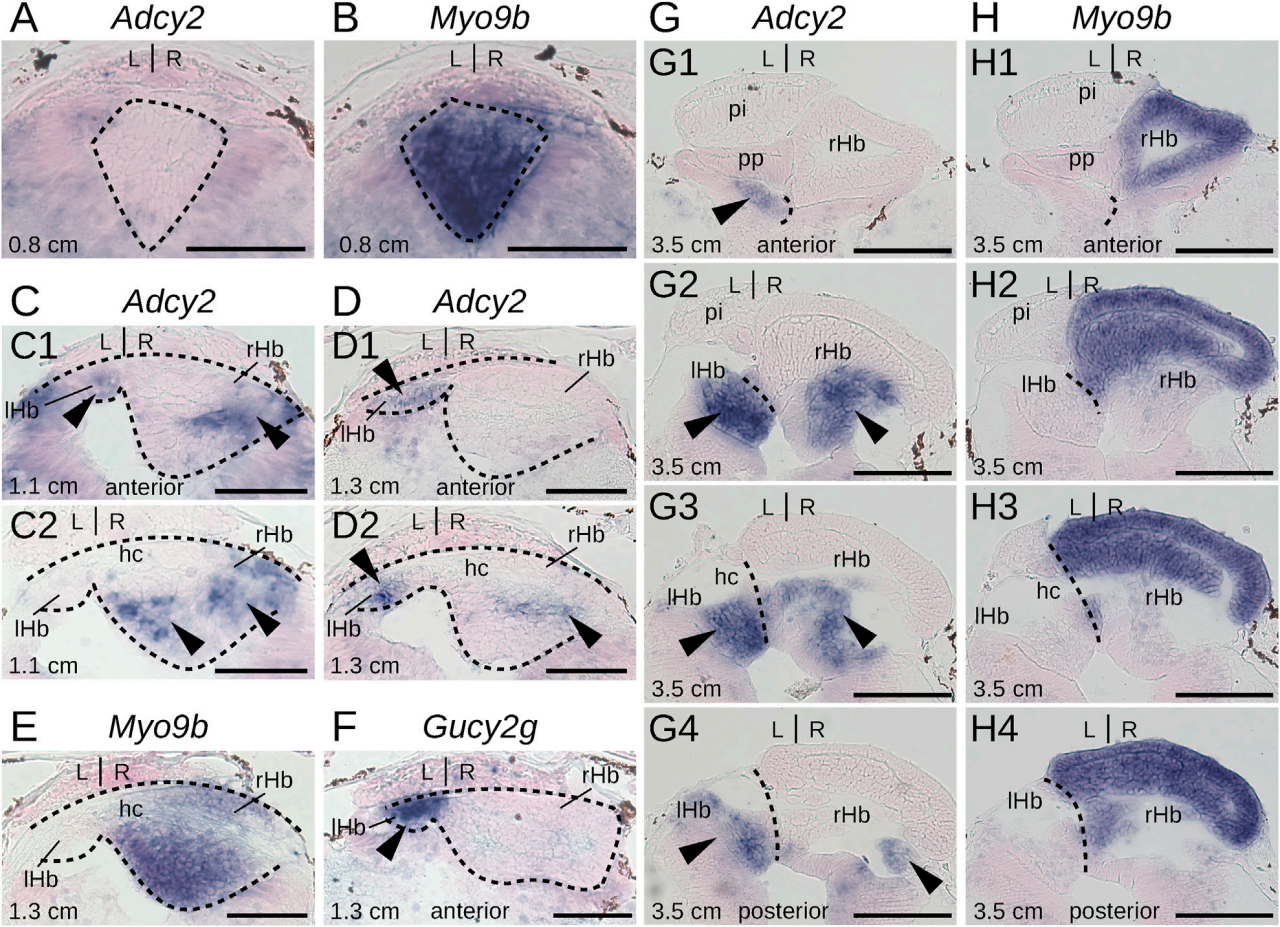
Elaboration of habenula subdomain organization during lamprey larval stages. **(A–H)** are transverse sections of developing habenulae in respectively 0.8 cm, 1.1 cm, 1.3 cm, and 3.5 cm lamprey larvae, after *in situ* hybridization with probes for *Adcy2* **(A,C,D, G)**, *Myo9b* **(B,E, H)** and *Gucy2g* **(F)**. **(A–F)** are from river lamprey specimens, **(G, H)** are from a sea lamprey specimen. **(A, B)** are from the same specimen, same for **(G, H)**. **(C1,D1,F)** are located anterior to the habenular commissure, **(C2,D2,E)** are located at the level of the anterior commissure **(E)** or immediately posterior to it **(C2,D2)**. **(G1–G4)** show successive sections from anterior to posterior, same for **(H1–H4)**. Dashed lines delimit habenulae in **(A–D)**. They delineate the boundary between left and right habenulae in **(G, H)**. Black arrowheads point to territories expressing *Gucy2g-* **(F)** and *Adcy2* **(C,D, G)**. Abbreviations: L, left; R, right; lHb, left habenula; rHb, right habenula; hc, habenular commissure. Scale bar = 50 μm in **(A–F)** and 100 μm in **(G, H)**.

To gain insights into the timing of neuronal identity elaboration in different habenular subdomains in the river lamprey, we analyzed the expression dynamics of four genes defining distinct territories of the dorsal right habenula (*Myo9b*, *Dgkb*, *Rab23* and *Prox1a*) and of two genes expressed, respectively, in the whole left and the ventral right habenula (*Adcy2*) and in a dorso-anterior subdomain of the left habenula (*Gucy2g*). ISH analysis of expression profiles was first carried out at prolarval stages (stages 26, 28, and 30) (Figure 6). No signal was observed for any of these markers at stage 26. At stage 28, all right-restricted markers show very similar signals in the right habenula, excluding ventral and posterior-most territories corresponding, by their location, to those of PCNA-positive neural progenitors (Figures 6A,C,E,G). These expression territories are maintained at stage 30 (Figures 6B,D,F,H). In contrast, no signal was detectable for either *Adcy2* or *Gucy2g* at these stages (not shown). Furthermore, no evidence for prolarval expression was obtained by analysis of a broader range of left-enriched genes (*Prkar2*, *Nwd2*, *Rock2*, and *Scgn*) (not shown). At larval stages, analysis was restricted to three gene markers, *Myo9b*, *Adcy2*, and *Gucy2g*. While *Adcy2* and *Gucy2g* expression is undetectable in 0.8 cm larvae, a major *Myo9b* signal is observable in the right habenula (Figures 7A,B). In contrast, *Gucy2g* and *Adcy2* expression is detectable in 1.1–1.3 cm larvae, in the left habenula anteriorly to the habenular commissure, with *Adcy2* being further expressed in the right habenula posteriorly to the habenular commissure (Figures 7C,D,F). In these larvae, *Myo9b* is broadly expressed in the right habenula (Figure 7E). In 3.5 cm larvae, the complementarity between the expression domains of *Myo9b* (dorsal) and *Adcy2* (ventral) becomes evident in the right habenula and *Adcy2* is detectable in the whole left habenula (Figures 7G,H). These expression characteristics are maintained in 7.0 cm larvae, which exhibit cell populations related, by their location, to those observed in adults (Supplementary Figures S7B,C). Thus, at this stage, the right ventral *Adcy2* territory, although continuous, contains both dense and more dispersed cell populations at locations comparable to those of the three *Adcy2*-positive subdomains in right adult habenulae (Supplementary Figures S7B1–4). In addition, expression of *Gucy2g* in 7.0 cm larvae becomes restricted to an anterior subdomain of the left habenula, as observed in adults (Supplementary Figure S7A).

### 3.6 Dynamics of Wnt activity in developing lamprey habenulae

**FIGURE 8:**
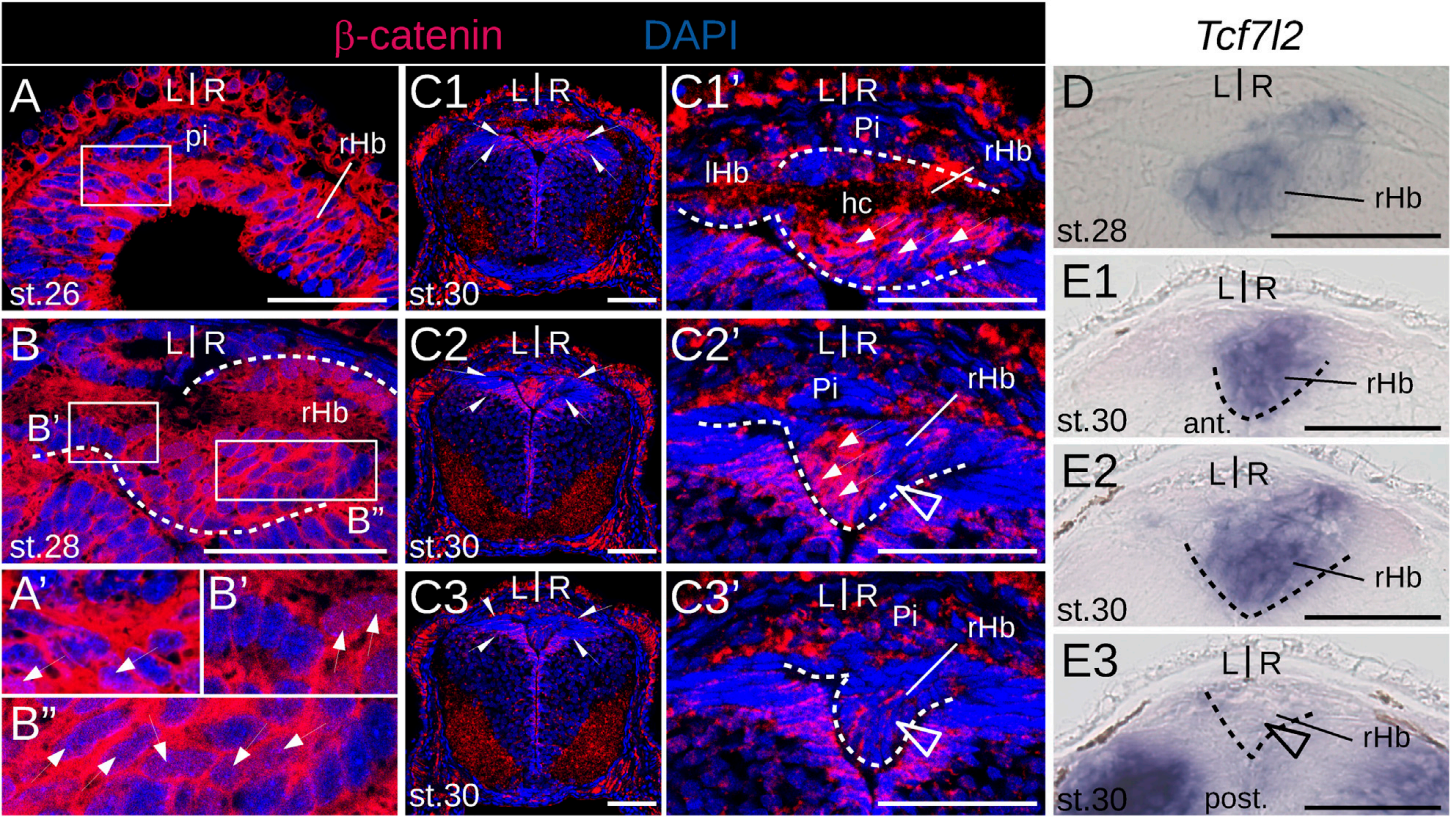
β−catenin and *Tcf7l2* expression in developing lamprey habenulae at prolarval stages. **(A, B)** and **(C1–C3)** are transverse sections of developing river lamprey habenulae at prolarval stages 26 **(A)**, 28 **(B)**, and 30 **(C1–C3)**, after immunohistochemistry with an antibody directed against β-catenin (red) an,d DAPI staining (blue). **(D)** and **(E1–E3)** are transverse sections of developing river lamprey habenulae at prolarval stages 28 **(A)** and 30 **(E1–E3)**, after *in situ hybridization* with a probe for *Tcf7l2*. **(C1–C3)** show sections of the same specimen from habenular commissure to posterior levels, **(E1–E3)** show sections of the same specimen from anterior to posterior. **(A′)**, **(B′,B′′)**, **(C1′, C3′)** are higher magnifications of the habenula region boxed respectively in **(A, B)**, **(C1–C3)**. Dashed lines delimit habenulae. Thin arrows point to nuclei positive for β-catenin. Empty arrowheads point to territories corresponding by their location to populations of PCNA-positive neural progenitors. Half-arrows in **(C1–C3)** delimit a thalamic neuroepithelial territory negative for β-catenin. Abbreviations: L, left; R, right; ant., anterior; post., posterior; lHb, left habenula; rHb, right habenula; hc, habenular commissure; pi, pineal field; ant., anterior; post., posterior; st., stage. Scale bar = 50 μm.

**FIGURE 9:**
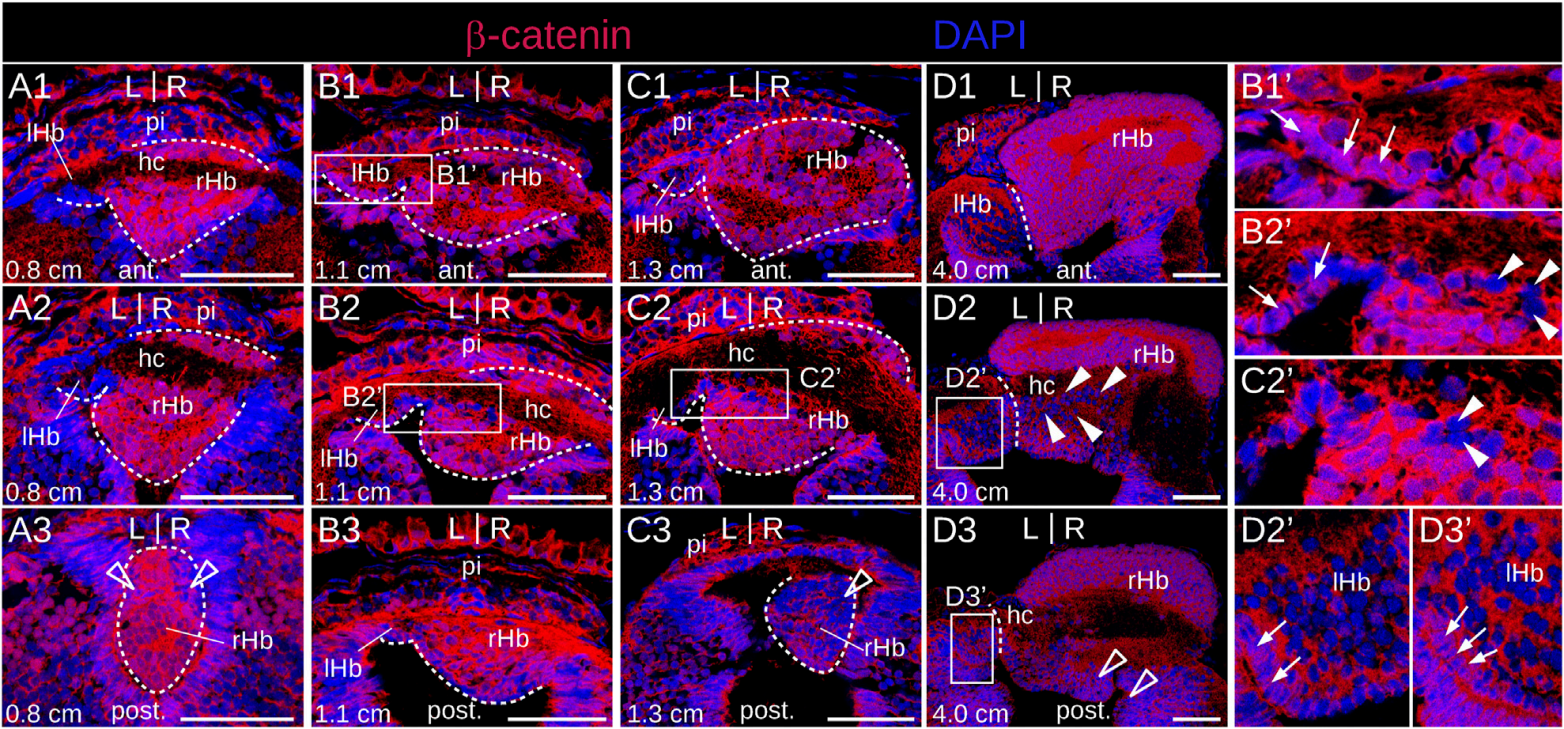
β−catenin and *Tcf7l2* expression in developing lamprey habenulae at larval stages. **(A1–A3)**, **(B1–B3)**, **(C1–C3)**, and **(D1–D3)** are transverse sections of developing habenulae in 0.8 cm, 1.1 cm, 1.3 cm and 4.0 cm lamprey larvae, after immunohistochemistry with an antibody directed against β-catenin (red) and DAPI staining (blue). **(A1–A3,B1–B3,C1–C3)** and **(D1–D3)** were from river lamprey and brook lamprey specimens respectively. **(A1–A3)** show sections of the same specimen from anterior to posterior, same for **(B1–B3)**, **(C1–C3)** and **(D1–D3)**. Dashed lines delimit habenulae in **(A1–A3)**, **(B1,B3)** and **(C1–C3)**. They delineate the boundary between left and right habenulae in **(D1–D3)**. **(B1′, B2′)**, **(C2′)**, **(D2′, D3′)** are higher magnifications of the habenula region boxed respectively in **(B1, B2)**, **(C2)**, **(D2, D3)**. Thin arrows point to nuclei positive for β-catenin in the ventricular zone of the left habenula. Empty arrowheads point to territories corresponding by their location to populations of PCNA-positive neural progenitors. White arrowheads point to nuclei negative for β-catenin, observed ventrally to the habenular commissure in 1.1–1.3 cm larvae. Abbreviations: L, left; R, right; ant., anterior; post., posterior; lHb, left habenula; rHb, right habenula; hc, habenular commissure; pi, pineal field; ant., anterior; post., posterior; st., stage. Scale bar = 50 μm.

Since Wnt signaling has been involved in the formation of habenulae asymmetry in catshark and zebrafish, we analyzed the activity profile of the Wnt pathway in developing lamprey habenulae at prolarval and larval stages, using IHC with an antibody directed against β−catenin (Figures 8A,B,C1–C3; Figure 9). An ISH analysis of *Tcf7l2*, whose expression is restricted to the right dorsal habenula in adults (Supplementary Figure S1M) was also included at prolarval stages (Figures 8DE1–E3). When habenulae become morphologically visible at stage 26, faint nuclear signals of β−catenin can be detected at dorsal habenula levels, but they remain absent from the adjacent pineal field (Figures 8A,A′). At stage 28, stronger nuclear signals are present in the right habenula, completely excluding the left habenula as well as ventral and posterior territories expressing PCNA-positive signals (Figures 8B,B′,B″). A similar profile is observed at stage 30, with widespread nuclear expression of β−catenin in the right habenula, except in neural progenitors, and with a total absence of nuclear signals in the left habenula (Figures 8C1–C3,C1′-C3′). Of note, at this stage, a sharp boundary demarcates territories either positive or negative for nuclear β−catenin in the ventricular pseudostratified neuroepithelium, which delimits the site of habenula formation (Figures 8C1–C3,C1′–C3′). No *Tcf7l2* signal is detected at stage 26 but a very specific expression is established at stages 28–30, in the same territories as those expressing other right dorsal markers and positive for nuclear β−catenin (Figures 8D,E1–E3). A related profile of β−catenin distribution is observed in 0.8 cm larvae, with strong nuclear signals in the right habenula, excluding PCNA-positive zones, and with an absence in the left habenula (Figures 9A1–A3). Two changes are observed in 1.1–1.3 cm larvae. While most nuclei maintain a strong β−catenin expression in the right habenula except at the posterior-most levels, that are positive for PCNA, a dispersed population of negative nuclei appears, ventrally to the habenular commissure (Figures 9B2,B2′,C2,C2′). In addition, while no nuclear β−catenin signal is detected in the left habenula at the prolarval stage 30 and in 0.8 cm larvae, some positive nuclei, interspersed with negative ones, are now present in ventricular cells (Figures 9B1–B3,C1–C3,B1′,B2′). These changes are confirmed in 4.0 cm larvae. In the right habenula, almost all cells exhibit strong nuclear β−catenin signals in dorsal *Myo9b*-positive territories. In contrast, the proportion of β−catenin-negative nuclei markedly increases in right ventral territories excluding ventricular zones, with a distribution reflecting *Adcy2*-positive territories (Figures 9D1–D3). In the left habenula, most nuclei are negative for β−catenin, except in the ventricular cell population, where nuclear PCNA signals are detectable (Figures 9D1–D3). In summary, we observe highly dynamic, asymmetric profiles of nuclear β−catenin localization both in neural progenitors and neuronal precursor subdomains.

## 4 Discussion

In this study, we have carried out an unbiased characterization of habenular asymmetries in lampreys and have compiled a detailed description of their formation during prolarval and larval stages. Our work provides strong support for the existence of a bipartite organization of the lamprey habenula, similar to that previously proposed based on a candidate gene approach with the catshark as reference (Lanoizelet et al., 2024). While the previously used gene markers (*Kctd12b* and *Prox1a/b*) showed overlapping territories of expression in the lamprey habenula, ISH analyses of genes retrieved from our transcriptomic analysis provide a much more detailed picture, as exemplified by the mutually exclusive expression patterns of *Myo9b* in the dorsal right habenula and of *Adcy2* in the left plus ventral right habenula, which, when taken together, cover the whole habenula. In line with an analysis of efferent projections from the lamprey habenula (Stephenson-Jones et al., 2012), our previous molecular characterization suggested that the partitioning observed in the lamprey reflects the organization into two subdomains of gnathostome habenulae (medial/lateral or dorsal/ventral in actinopterygians). The present analysis, which expands the set of territory markers available in the lamprey, allowed us to identify additional genes that further support the evolutionary relationship between the left plus ventral right habenulae of lampreys and the medial habenulae of jawed vertebrates (Table 1). Of the 8 lamprey genes with broad expression in these territories, 4 (*LfNecab1*, *Prkar2a*, *Gpr26*, and *Nwd2*) possess one ohnolog in the mouse with expression that is largely restricted to the medial habenula (Chen et al., 2022; Lein et al., 2007; London et al., 2020): *MmNecab2*, *MmPrkar2a*, *MmGpr26*, and *MmNwd2* (Supplementary Figure S8). These genes may therefore correspond to ancient and highly conserved markers of the medial habenular territory. We also identified novel habenular subdomains, both on the left and on the right side. While the left and the ventral right habenulae appear molecularly related, the left habenula contains a dorsal and anterior subdomain expressing at least two genes (*Gucy2g* and *Scgn*), whose expression is undetectable on the right side. Such an asymmetric partitioning of the medial habenula has previously been reported both in the zebrafish and the catshark (Aizawa et al., 2007; deCarvalho et al., 2014; Gamse et al., 2005; 2003; Lanoizelet et al., 2024; Pandey et al., 2018), but the limited number of markers available does not allow any firm conclusions as to possible cross-species relationships. Similarly, in the dorsal right habenula, *Rab23* defines a lateral subdomain without any clear correspondence in other species. These difficulties to detect cross-species similarities in subdomain architecture and asymmetry of habenulae may be related to lineage-specific diversifications, as recently reported in teleosts and tetrapods (Lanoizelet et al., 2024), or simply due to the scarcity of available data, precluding a robust phylogenetic approach. Spatially-resolved, genome-wide characterizations of habenulae from a variety of different species will thus be crucial for obtaining a more comprehensive view of the conservation and divergence of habenular organization across vertebrates.

**TABLE 1:** Dynamics of expression of subdomain markers in lamprey habenulae at prolarval and larval stages**.** For each of the three markers analyzed (*Myo9b*, *Adcy2*, and *Gucy2g*) and at each stage studied, “−” and “+” respectively indicate the absence and presence of expression in developing habenulae. When expression was detected, the labeled territory is indicated. Abbreviations: lHb, left habenula; rHb, right habenula; st., stage.

	Prolarva			Larva			
	st.26	st.28	st.30	0.8 cm	1.1 cm	1.3 cm	3.5–7.0 cm
*Myo9b*	-	+ rHb	+ rHb	+ rHb	+ rHb	+ rHb	+ dorsal rHb
*Adcy2*	-	-	-	-	+ lHb, rHb	+ lHb, rHb	+ lHb, ventral rHb
*Gucy2g*	-	-	-	-	+ lHb	+ lHb	+ anterior lHb

**FIGURE 10:**
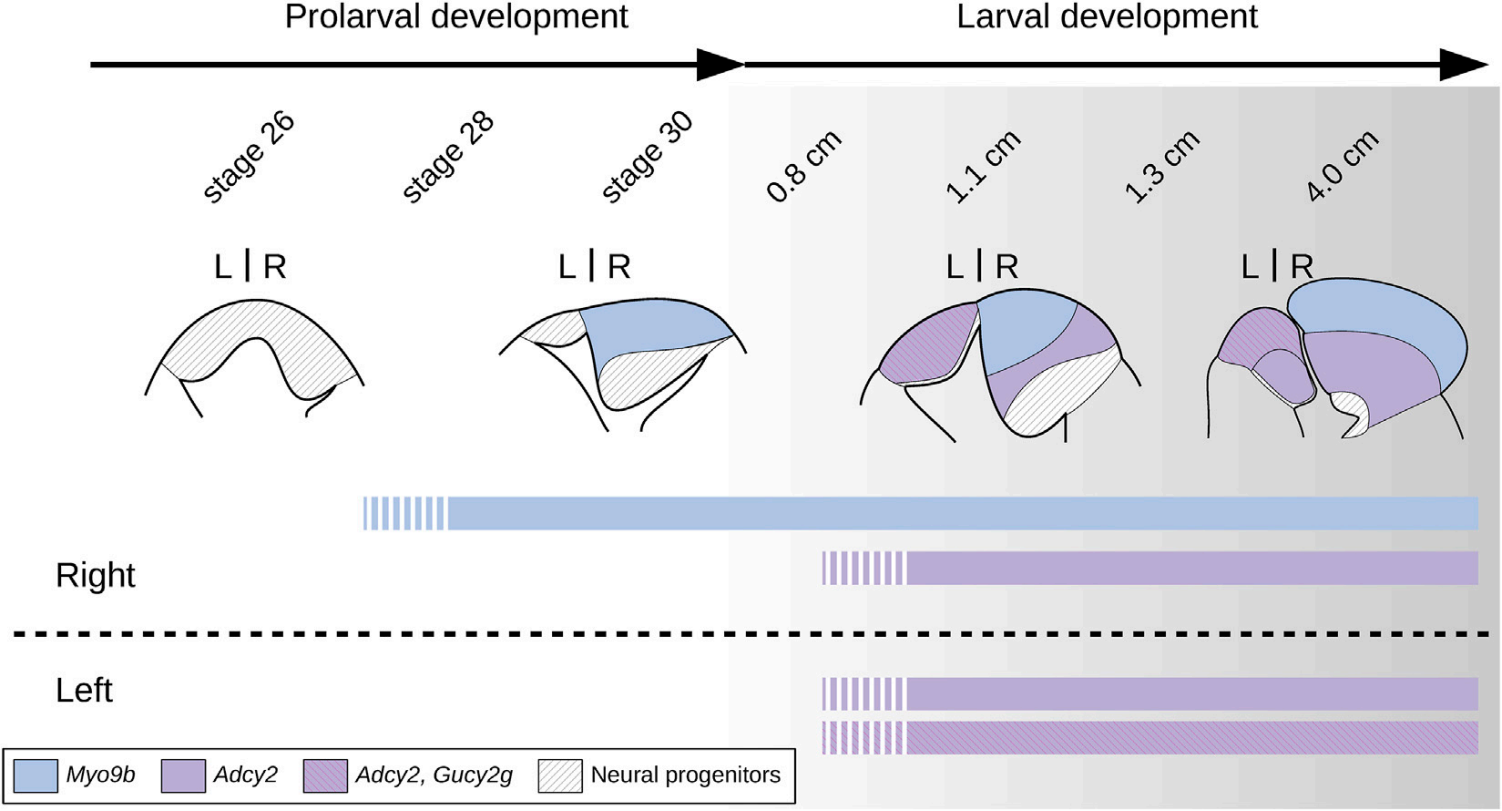
Scheme showing major changes in the organization of lamprey habenulae during prolarval and larval development. Broad transitions in the general organization of habenulae are shown with the *Myo9b*-positive right dorsal habenula in blue and with the bilateral, *Adcy2-*positive territory spanning both the left and ventral right habenula in purple, and its *Gucy2g*-positive subdomain hatched (magenta lines on purple background). Territories of neural progenitors are shown hatched (black lines on white background). The developmental stages shown in the diagram do not show a homogeneous developmental progression but have been chosen based on the progression of habenula formation and of the establishment of habenular asymmetries. At stage 26, no indication of neuronal identity elaboration is observed. Starting from prolarval stage 28 and until at least early larval stages (0.8 cm in the river lamprey), the right habenula expands and expresses neuronal signatures of the adult dorsal right habenula. In contrast, the left and ventral right habenulae do not markedly expand and exhibit no indication of neuronal identity elaboration until significantly later larval stages (1.1 cm in the river lamprey). At the temporal resolution of our analysis, the expression initiation of *Gucy2g*, a marker for a dorso-anterior subdomain of the left habenula, was synchronous with that of *Adcy2* in ventral territories of left and right habenulae. However, we cannot exclude differential temporal regulations of neuronal identity elaboration between these *Gucy2g*-positive and negative subdomains.

From a developmental perspective, we previously reported major size differences between the left and the right habenulae of lampreys, starting from the earliest stages of habenula formation (Lagadec et al., 2015). The availability of subdomain markers leads to a more comprehensive picture of these size asymmetries and provides new insights into the mode of habenula development in the lamprey (Figure 10). We found that the first habenula subdomain to form, during prolarval and the earliest larval stages, is the dorsal right one, and that this right-restricted process is mainly responsible for shaping the left/right size asymmetry we previously observed. Based on the temporal resolution of our analysis, the left plus ventral right habenulae form simultaneously, and significantly later (in larvae larger than 1.0 cm in size) as molecularly related bilateral territories. However, a more extensive sampling of time points during the prolaval-to-larval transition will be necessary to more precisely define the progression of habenular development during this period. In the absence of such data, a preliminary conclusion is that the temporal regulation of subdomain formation in the lamprey habenula is reminiscent of the successive formation, in the catshark, of territories respectively related to the lateral and medial habenulae on the right side (Lanoizelet et al., 2024). A major difference between the two species is the absence, in the lamprey, of a habenular territory of lateral identity on the left side, an asymmetry established at early stages of habenula formation in the catshark. The underlying cellular mechanisms remain to be assessed. We found no evidence for apoptosis at the prolarval stages studied, but we cannot exclude that some apoptotic cells may have escaped detection, either in micro-domains or at time points not included in this study. Newly born, HuC/D-positive neurons were detected both on the right and the left sides starting from early stages of habenula formation, without clear evidence for an asymmetric temporal regulation of cell cycle exits (Aizawa et al., 2007; Guglielmi et al., 2020; Lagadec et al., 2018; Powell et al., 2024; Lagadec et al., 2024) or for an earlier onset of neuronal differentiation on the left as observed in gnathostomes (Roussigné et al., 2009). However, the involvement of an asymmetric regulation of neurogenesis should be further assessed and quantified using analyses of cell cycle exits during the elaboration of habenular subdomains. Finally, an asymmetric regulation of cell proliferation, as reported in the catshark (Lagadec et al., 2018), may be a major factor in the development of size asymmetries in lamprey habenulae. In line with this hypothesis, only dispersed PCNA-positive cells were observed on the left side from late prolarval stages (stage 29) to relatively advanced larval stages, while a dense posterior population of neuroepithelial cells was maintained on the right side, evoking a major, localized proliferation center. Quantitative analyses of proliferation rates will be crucial to test this possibility. Concerning the *Adcy2*-positive left and ventral right subdomains, the similar timing of their formation does not seem to translate into a symmetry of their neuronal identities, as shown by the presence of a left-restricted *Gucy2g*-positive anterior subterritory. In the zebrafish, the asymmetric partitioning of the medial habenula, their putative gnathostome counterpart, results from an asymmetric temporal regulation of neurogenesis, also described in the developing medial habenula of the catshark, although a causal relationship between the processes observed in zebrafish, catshark, and lamprey remains to be demonstrated (Aizawa et al., 2007; Guglielmi et al., 2020; Lagadec et al., 2018; Powell et al., 2024; Lagadec et al., 2024). Detailed analyses of the timing of subterritory-specific cell cycle exits will have to be carried out in the lamprey left habenula to address the possible conservation of this process. As in the zebrafish (Concha et al., 2003; Gamse et al., 2003; Lekk et al., 2019), it might involve spatial, left- and anterior-restricted cues, possibly resulting from interactions with the parapineal, which, in the lamprey, is located in close proximity to the initial expression domain of *Gucy2g*.

We previously proposed an ancient involvement of Wnt signaling in habenular asymmetry formation based not only on the role of the Wnt pathway in the catshark and in the zebrafish, but also on asymmetric profiles of nuclear β−catenin distribution in habenulae at advanced stage of differentiation in a broad sampling of species, including the river lamprey (Lanoizelet et al., 2024). The dynamic and highly asymmetric profiles of nuclear β−catenin in developing lamprey habenulae confirm and extend this hypothesis. They indeed suggest that asymmetric Wnt activity concerns PCNA-positive neural progenitors, as well as neuronal subdomain precursors, as identified by signature markers. The strong nuclear β−catenin signals, initiated in the *Myo9b*-/*Prox1a/b-*positive right dorsal habenula concomitantly with the onset of *Myo9b*-/*Prox1a/b* expression and maintained throughout development, evoke a conserved role for Wnt signaling in promoting the corresponding neuronal identity: lateral right in ancestral gnathostomes, as exemplified in the catshark (Lanoizelet et al., 2024), dorsal right in the lamprey. The absence of nuclear β−catenin in neural progenitors of the right habenula of prolarvae and early larvae is also consistent with a regulation operating in post-mitotic precursors, as reported in the catshark (Lanoizelet et al., 2024). In contrast, the nuclear distribution of β−catenin signals appeared highly dynamic in the left habenula, with positive signals coinciding with PCNA-positive cell populations in larvae larger than 1.1 cm in size. This is consistent with an involvement of Wnt signaling in the control of neurogenesis, as demonstrated in the zebrafish (Guglielmi et al., 2020; Powell et al., 2024). Altogether, these data suggest that the lamprey may have retained distinct, ancient roles of Wnt signaling in habenular asymmetry formation, which remain to be directly addressed using experimental perturbations of Wnt activity. Pharmacological approaches combined with CRISPR-Cas9 inactivation of genes coding for Wnt pathway components and regulators as reported in the lamprey (Square et al., 2015) will thus be crucial to test this hypothesis and to obtain a comprehensive view of the evolution of Wnt functions during habenular asymmetry formation. In conclusion, the unique characteristics of habenula asymmetry formation in lampreys, together with the rise of advanced technologies applicable to these species such as spatial transcriptomics and gene editing, substantiate the interest of this taxon for comparative studies and allow new perspectives on the developmental logic underlying the recurrence and variation of habenular asymmetries across vertebrates.

## Data Availability

The datasets presented in this study can be found in online repositories (NCBI). The names of the repository/repositories and accession number(s) can be found in the article/Supplementary Material (Supplementary Table S2, S3).
