# Supplementary material for: The lamprey habenula provides an extreme example for the temporal regulation of asymmetric development
Source: Front Cell Dev Biol. 2025 Feb 6;13:1528797. doi: 10.3389/fcell.2025.1528797 (PMC11839670; doi:10.3389/fcell.2025.1528797)
Supplement: Supplementary file 3 [file DataSheet1.pdf]

The lamprey habenula provides an extreme example for the temporal regulation of asymmetric development

SUPPLEMENTARY MATERIAL

List of Supplementary Figures and Tables:

Supplementary Figure S1. Transcriptomic analysis of asymmetries in adult lamprey habenulae.

Supplementary Figure S2. Details of expression profiles of *Gucy2g*, *Adcy2*, *Myo9b* and *Rab23* in adult lamprey habenulae.

Supplementary Figure S3. Morphology of developing habenulae in stage 30 prolarvae and 6.2 cm larvae.

Supplementary Figure S4. Analysis of apoptosis in developing habenulae of stage 26, 28 and 29 prolarvae.

Supplementary Figure S5. Neuronal differentiation in developing habenulae of stage 26 prolarvae.

Supplementary Figure S6. Details of proliferation-differentiation patterns in developing habenulae.

Supplementary Figure S7. Habenula subdomain organization in 7.0 cm lamprey larvae.

Supplementary Figure S8. Expression of mouse ohnologs of lamprey subdomain markers.

Supplementary Table S1. Transcriptomic reference database used for read mapping in the river lamprey.

Supplementary Table S2. Datasets used to generate a transcriptomic reference in the river lamprey.

Supplementary Table S3. River lamprey sequences used as probes in *in situ hybridization*.

Supplementary Table S4. Primary antibodies used.

Supplementary Table S5. List of differentially expressed genes obtained in the transcriptomic comparison of left versus right habenulae of adult river lampreys.

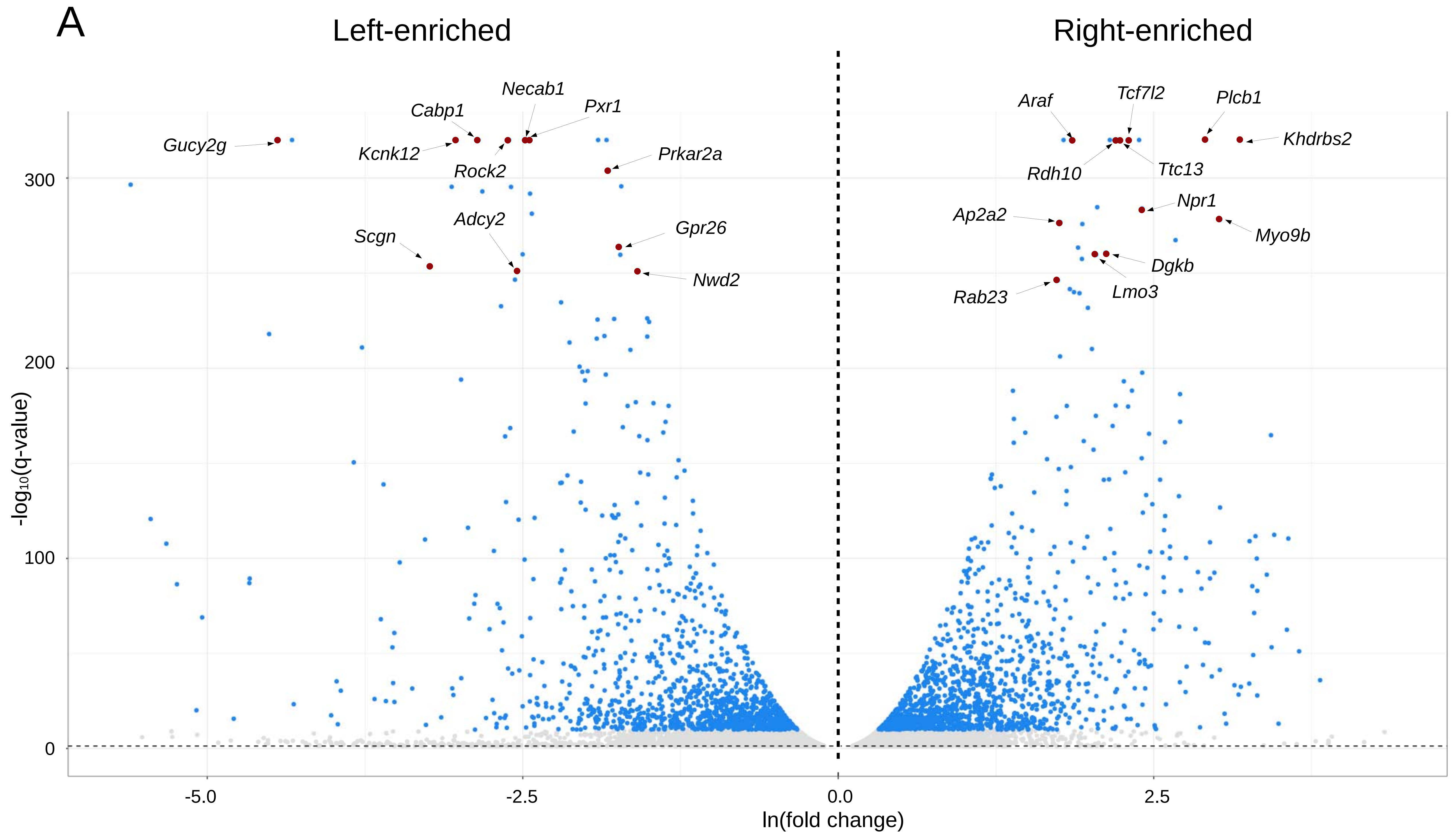

Supplementary Figure S1 (continued on next page)

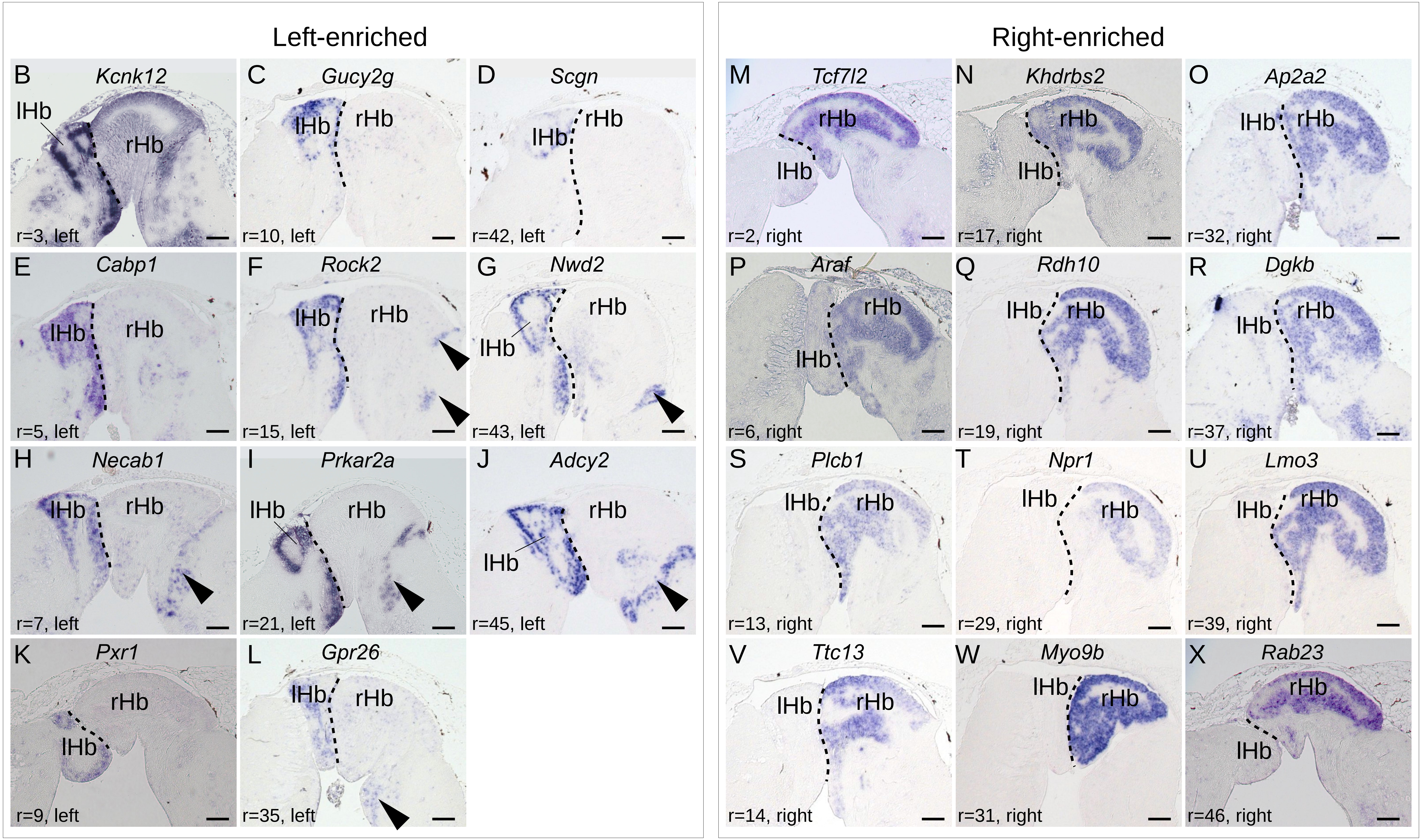

Supplementary Figure S1 (continued)

See legend on next page

Supplementary Figure S1. Transcriptomic analysis of asymmetries in adult lamprey habenulae. (A) shows a volcano plot of differentially expressed genes between the left and the right habenula. Each dot represents a differentially expressed gene, with left- and right-enriched genes shown on the left and right sides of the graph respectively. Red dots indicate genes further analyzed by *in situ* hybridization. **(B-X)** show transverse sections of adult river lamprey habenulae after *in situ* hybridization with probes for *Kcnk12* (B), *Gucy2g* (C), *Scgn* (D), *Cabp1* (E), *Rock2* (F), *Nwd2* (G), *Necab1* (H), *Prkar2a* (I), *Adcy2* (J), *Pxr1* (K), *Gpr26* (L), *Tcf7l2* (M), *Khdrbs2* (N), *Ap2a2* (O), *Araf* (P), *Rdh10* (Q), *Dgkb* (R), *Plcb1* (S), *Npr1* (T), *Lmo3* (U), *Ttc13* (V), *Myo9b* (W), and *Rab23* (X). Profiles shown in (B-L) and (M-X) were obtained for left- and right-enriched genes respectively. For each gene, its rank (r) in the list of differentially expressed genes ordered by decreasing statistical support is indicated at the bottom left of the *in situ* hybridization profile, together with its side of enrichment. Dashed lines show the boundary between left and right habenulae. Abbreviations: L, left; R, right; lHb, left habenula; rHb, right habenula. Scale bar=100 µm.

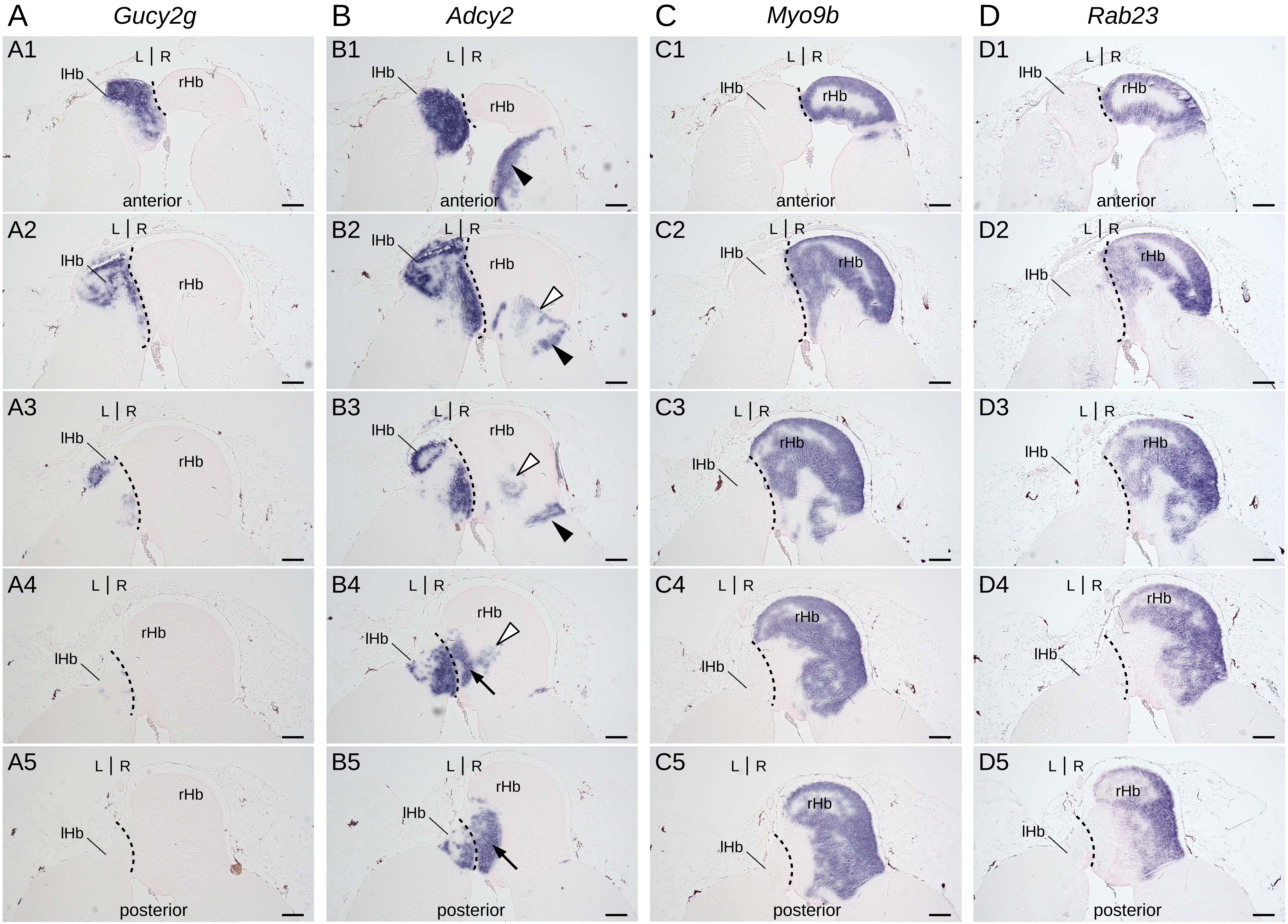

Supplementary Figure S2

See legend on next page

Supplementary Figure S2. Details of expression profiles of *Gucy2g*, *Adcy2*, *Myo9b*, and *Rab23* in adult lamprey habenulae. (A,B,C,D) show transverse sections of adult river lamprey habenulae after *in situ* hybridization with probes for *Gucy2g* (A), *Adcy2* (B), *Myo9b* (C), and *Rab23* (D). All sections were from the same specimen. (A1) to (A5) show successive sections from anterior to posterior, same for (B1) to (B5), (C1) to (C5), and (D1) to (D5). Dashed lines show the boundary between left and right habenulae. Thin arrows as well as black and white arrowheads point to discrete ventral subterritories expressing *Adcy2* in the right habenula. Abbreviations: L, left; R, right; lHb, left habenula; rHb, right habenula. Scale bar=100 μm.

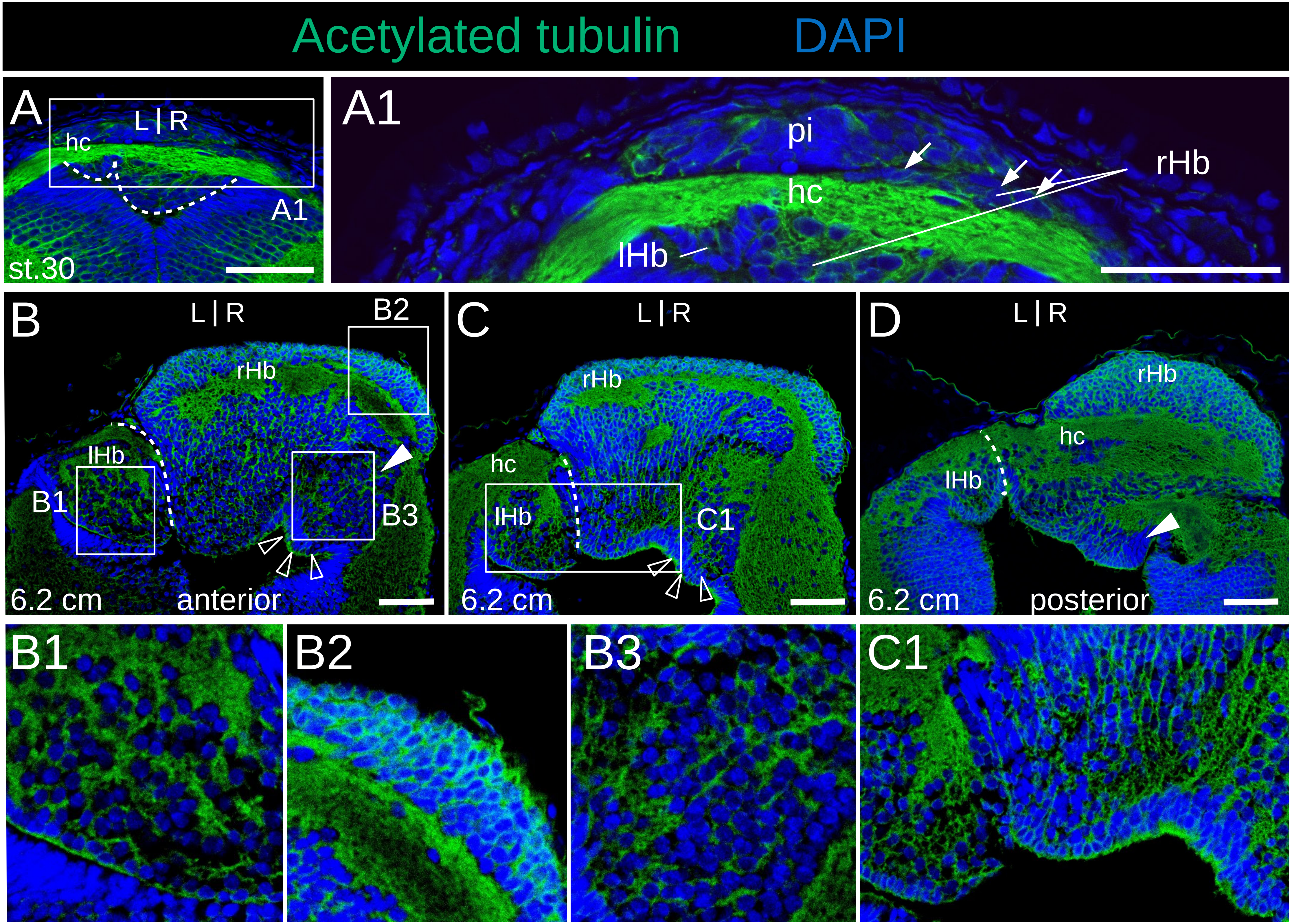

Supplementary Figure S3

See legend on next page

Supplementary Figure S3. Morphology of developing habenulae in stage 30 prolarvae and 6.2 cm larvae. (A) and **(B,C,D)** respectively show transverse sections of developing habenulae of a stage 30 river lamprey prolarva (A) and of a 6.2 cm sea lamprey larva (B-D), after immunohistochemistry with an antibody directed against acetylated tubulin (green) and DAPI staining (blue). (A) shows the same specimen as Figure 2F1-F4, with a higher magnification of the boxed region at the level of the habenular commissure in (A1). (B), (C), and (D) show sections of the same specimen from anterior to posterior. (B1-B3) and (C1) show higher magnifications of the habenula regions boxed respectively in (B) and (C). Dashed lines delimit the forming habenula in (A), the left habenula being restricted to a thin cell population in contact with the habenular commissure at this stage. Dashed lines delineate the boundary between the left and the right habenula in (B), (C), and (D). Thin arrows in (A1) point to a territory of the right habenula located dorsally to the habenular commissure. Empty arrowheads in (B,C) indicate a second lobe in the right habenula, a white arrowhead in (C) points to a zone exhibiting the morphology of a pseudostratified neuroepithelial. Abbreviations: L, left; R, right; lHb, left habenula; rHb, right habenula; hc, habenular commissure; pi, pineal field; st., stage. Scale bar=50 μm in (A,A1) and 100 μm in (B-D).

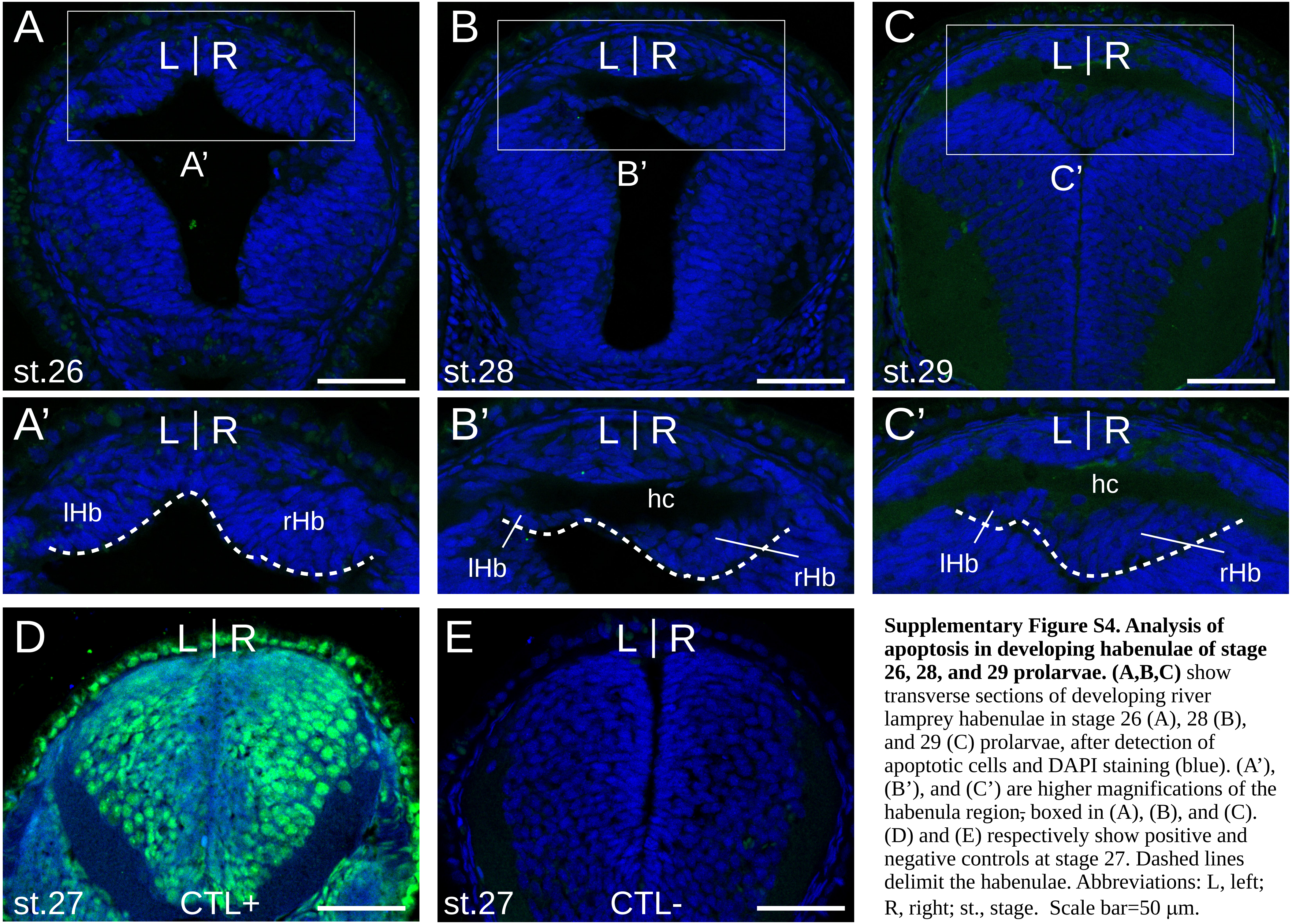

Supplementary Figure S4. Analysis of apoptosis in developing habenulae of stage 26, 28, and 29 prolarvae. (A,B,C) show transverse sections of developing river lamprey habenulae in stage 26 (A), 28 (B), and 29 (C) prolarvae, after detection of apoptotic cells and DAPI staining (blue). (A'), (B'), and (C') are higher magnifications of the habenula region, boxed in (A), (B), and (C). (D) and (E) respectively show positive and negative controls at stage 27. Dashed lines delimit the habenulae. Abbreviations: L, left; R, right; st., stage. Scale bar=50 µm.

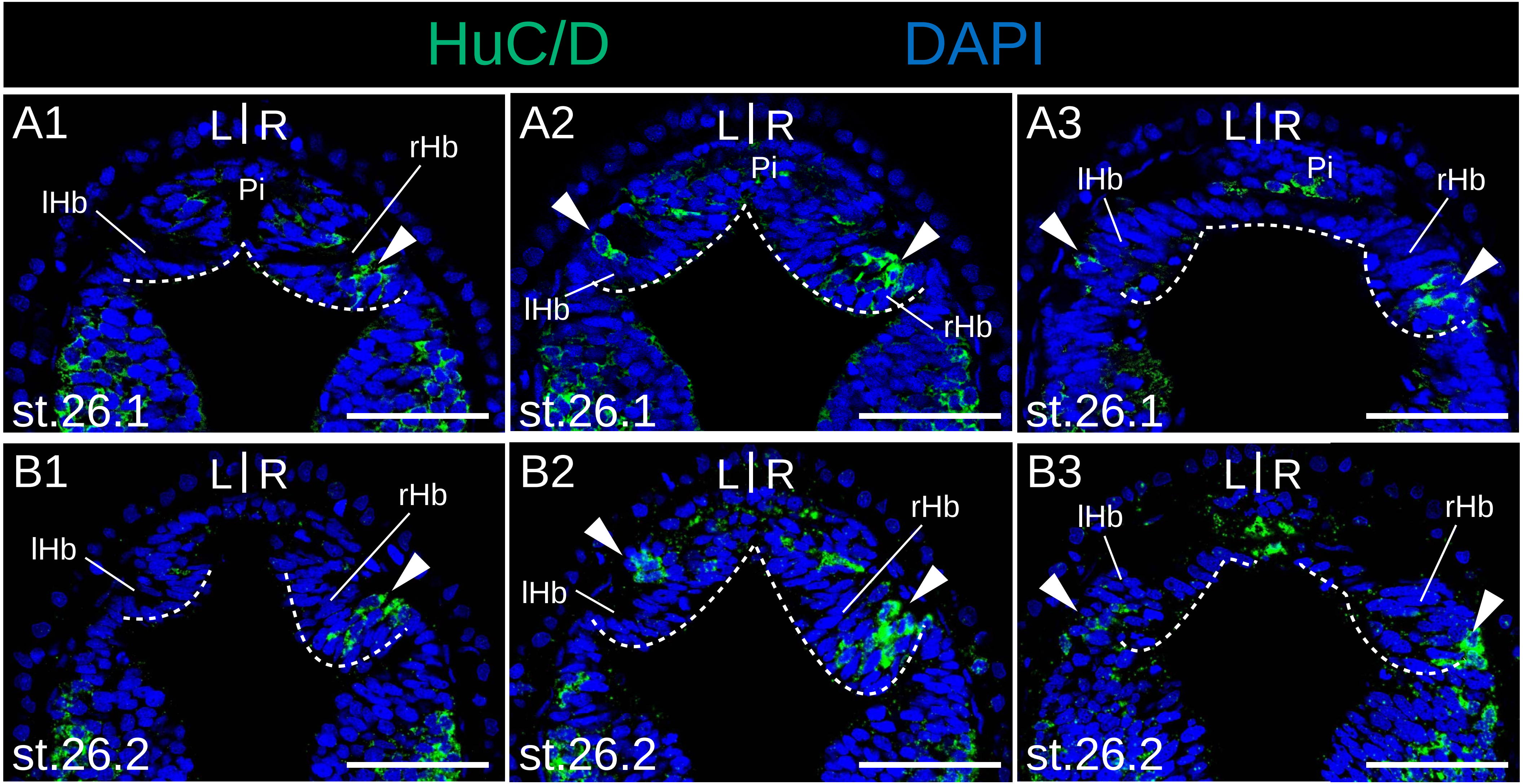

Supplementary Figure S5

See legend on next page

Supplementary Figure S5. Neuronal differentiation in developing habenulae of stage 26 prolarvae. (A1-A3) and **(B1-B3)** show transverse sections of developing river lamprey habenulae in two distinct stage 26 prolarvae, after immunohistochemistry with an antibody directed against HuC/D (green) and DAPI staining (blue). The specimens shown are different from the one shown in Figure 4B. (A1) to (A3) show successive sections from anterior to posterior, same for (B1) to (B3). Dashed lines delimit the habenulae. White arrows point to HuC/D-positive cells. Abbreviations: L, left; R, right; st., stage. Scale bar=50 μm.

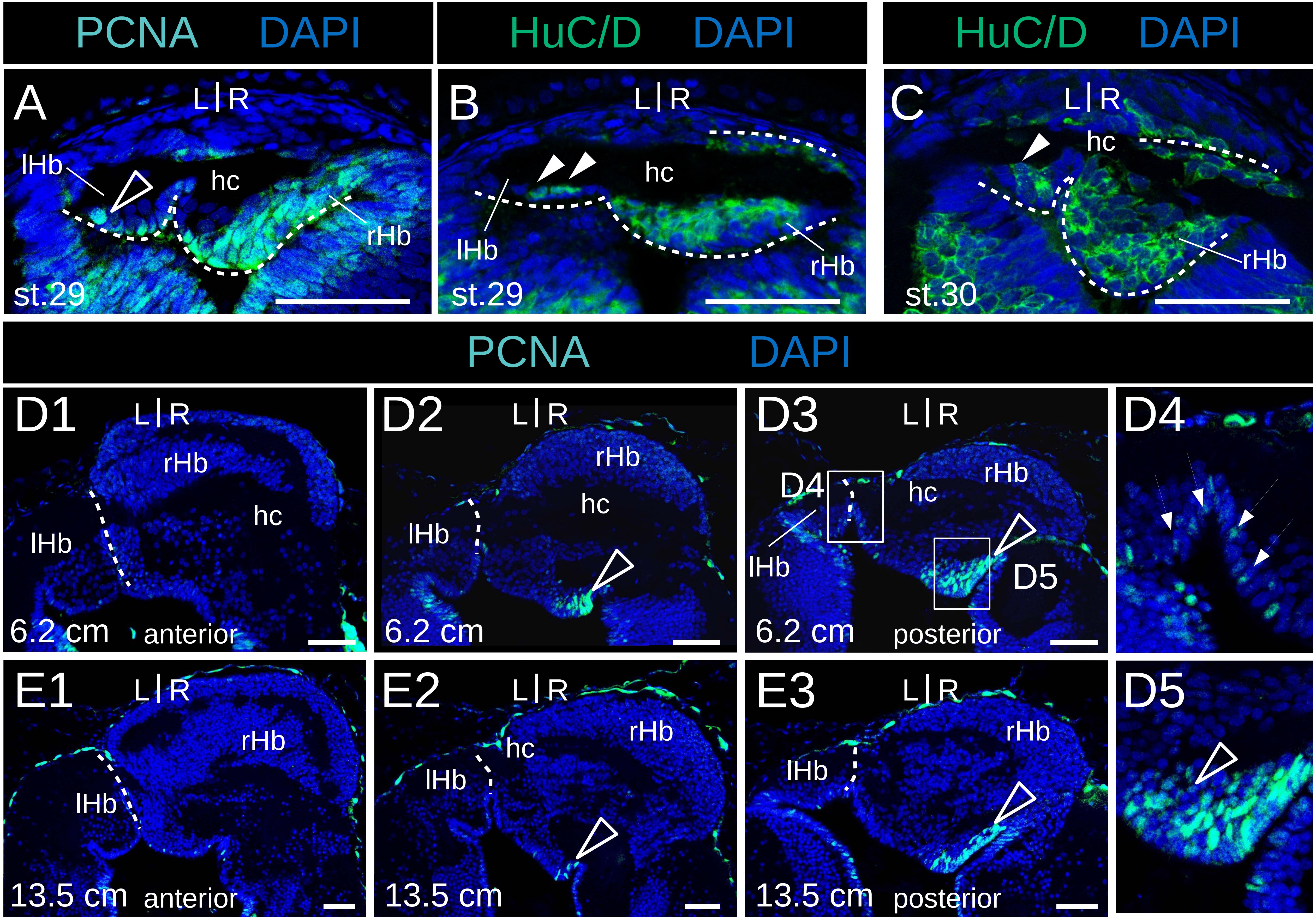

Supplementary Figure S6

See legend on next page

Supplementary Figure S6. Details of proliferation-differentiation patterns in developing habenulae. (A-C) show transverse sections of habenulae in stage 29 (A,B) and stage 30 river lamprey prolarvae, after immunohistochemistry with antibodies directed against PCNA (green) (A), HuC/D (green) (B,C), and DAPI staining (blue). **(D1-3)** and **(E1-3)** respectively show sections of 6.2 cm and 13.5 cm sea lamprey larvae. (D1), (D2), and (D3) show successive sections from anterior to posterior, same for (E1), (E2), and (E3). (D4) and (D5) show higher magnifications of the habenula regions boxed in (D3). Dashed lines delimit the habenulae in (A-C). Dashed lines delineate the boundary between the left and the right habenula in (D1-3,E1-3). White arrows in (B,C) point to HuC/D-positive cells in the left habenula of stage 29 and stage 30 prolarvae. Empty arrows point to PCNA-positive cells located at ventricular levels of the left habenula in (A) and in a restricted ventral and posterior cell population of the right habenula in (D2,D3,E2,E3). Thin arrows show faint PCNA signals in dispersed ventricular cells in the left habenula or at medial levels in the right habenula (D4). Abbreviations: L, left; R, right; hc, habenular commissure; st., stage. Scale bar=50 μm.

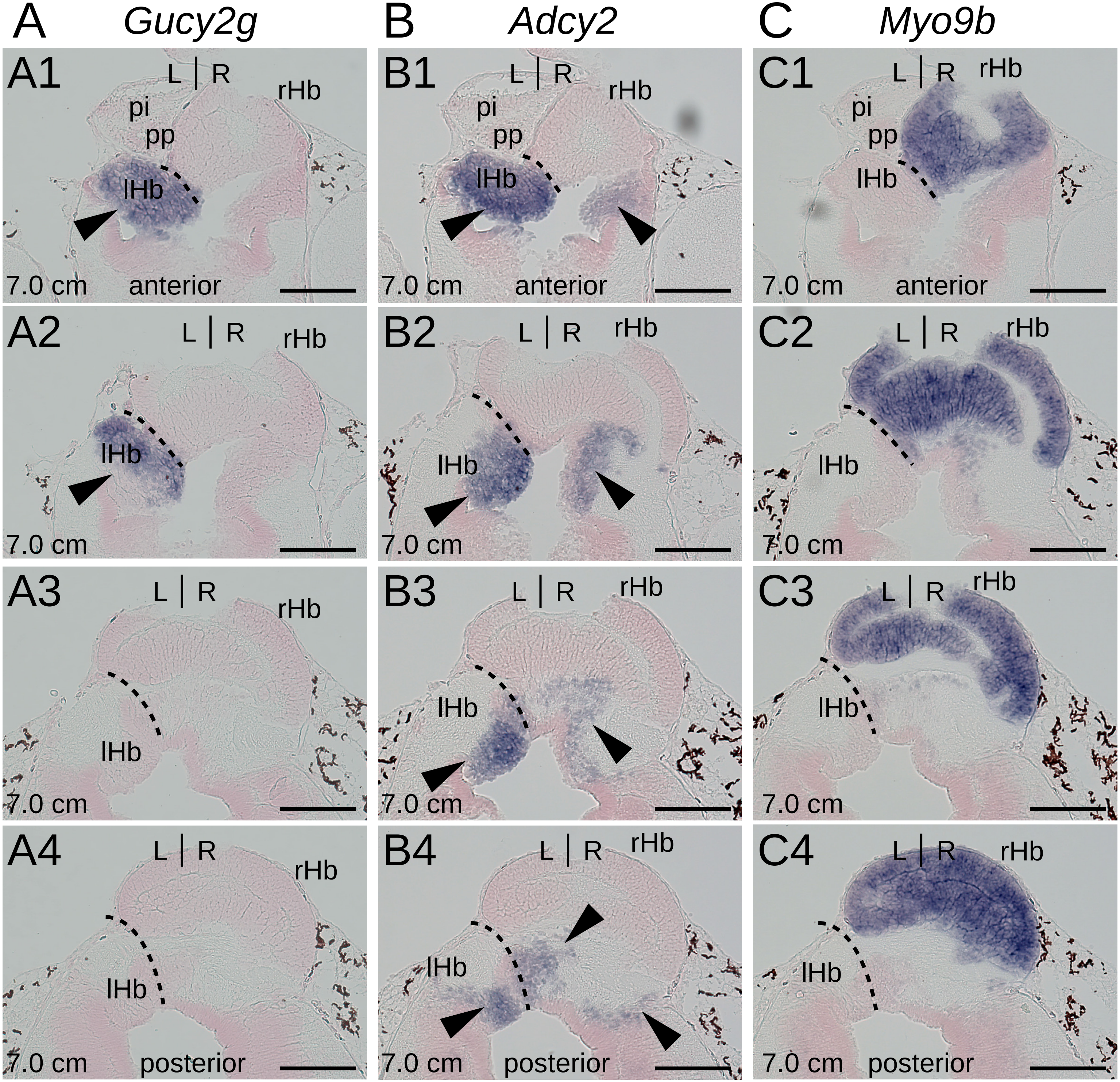

Supplementary Figure S7

See legend on next page

Supplementary Figure S7. Habenula subdomain organization in 7.0 cm lamprey larvae. (A), (B), and **(C)** show transverse sections of habenulae in 7.0 cm sea lamprey larvae, after *in situ* hybridization with probes for *Gucy2g* (A), *Adcy2* (B), and *Myo9b* (F). All sections are from the same specimen. (A1) to (A4) show successive sections from anterior to posterior, same for (B1) to (B4), and for (C1) to (C4). Dashed lines delineate the boundary between left and right habenulae. Black arrowheads point to territories expressing *Gucy2g* (A) and *Adcy2* (B). Abbreviations: L, left; R, right; lHb, left habenula; rHb, right habenula; hc, habenular commissure; pi, pineal; pp, parapineal. Scale bar=100 μm.

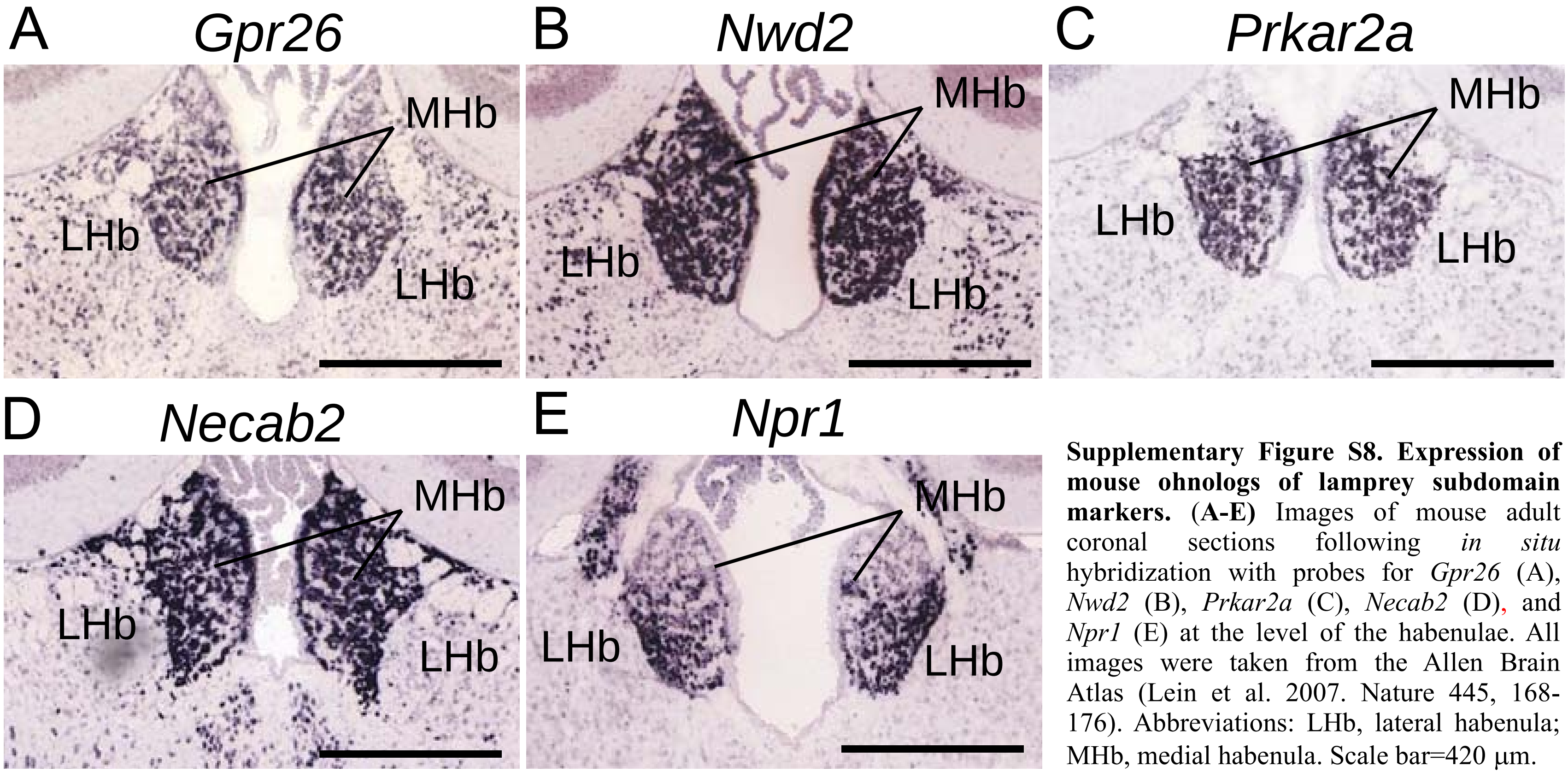

Supplementary Figure S8. Expression of mouse ohnologs of lamprey subdomain markers. **(A-E)** Images of mouse adult coronal sections following *in situ* hybridization with probes for *Gpr26* (A), *Nwd2* (B), *Prkar2a* (C), *Necab2* (D), and *Npr1* (E) at the level of the habenulae. All images were taken from the Allen Brain Atlas (Lein et al. 2007. Nature 445, 168-176). Abbreviations: LHb, lateral habenula; MHb, medial habenula. Scale bar=420 µm.

Supplementary Table S1. Transcriptomic reference database used for read mapping in the river lamprey. The file lists the gene clusters with their sequences in fasta format, obtained as described in Materials and methods section 2.3.

Supplementary Table S2. Datasets used to generate a transcriptomic reference in the river lamprey. Columns A, B and C respectively contain the NCBI identifiers for the datasets, their tissue from which thery were generated and the corresponding read number.

Supplementary Table S3. River lamprey sequences used as probes in *in situ hybridization*. Columns A, B and C respectively contain gene names, the corresponding cluster in the reference database and the corresponding NCBI protein identifier. For each gene, its rank in the list of asymmetrically expressed genes identified by the transcriptomic analysis and ordered by increasing q-values is indicated in column D, together with its expression laterality (yellow, left-enriched genes; blue, right-enriched genes). Annotations, obtained by blastx or blastn against the genome of *Lethenteron reissneri*, are indicated respectively in columns E and G, with the identifier of *L.reissneri* protein when available in F. Column H indicates the panel where the *in situ hybridization* profile is shown in Figure S1 and column I provides the sequence used as probe.

Supplementary Table S4. Primary antibodies used. Column A, B, C and D respectively indicate the antigen against which the primary antibody is directed, its type (monoclonal or polyclonal), its commercial reference and the working dilution used. Columns E, F and G respectively indicate the secondary antibody used, its commercial reference and the working concentration used.

Supplementary Table S5. List of differentially expressed genes obtained in the transcriptomic comparison of left versus right habenulae of adult river lampreys. Columns A and B respectively indicate the gene cluster identified, its annotation. p-values and q-values are shown in columns C and D, fold change in column F. The gene clusters are ranked by increasing q-values. In column A, clusters enriched on the left side are shaded in yellow, those enriched on the right side in blue.
